# High-Throughput Ligand Discovery Reveals a Sitewise Gradient of Diversity in Broadly Evolved Hydrophilic Fibronectin Domains

**DOI:** 10.1371/journal.pone.0138956

**Published:** 2015-09-18

**Authors:** Daniel R. Woldring, Patrick V. Holec, Hong Zhou, Benjamin J. Hackel

**Affiliations:** Department of Chemical Engineering and Materials Science, University of Minnesota, Minneapolis, MN, United States of America; University of Edinburgh, UNITED KINGDOM

## Abstract

Discovering new binding function via a combinatorial library in small protein scaffolds requires balance between appropriate mutations to introduce favorable intermolecular interactions while maintaining intramolecular integrity. Sitewise constraints exist in a non-spatial gradient from diverse to conserved in evolved antibody repertoires; yet non-antibody scaffolds generally do not implement this strategy in combinatorial libraries. Despite the fact that biased amino acid distributions, typically elevated in tyrosine, serine, and glycine, have gained wider use in synthetic scaffolds, these distributions are still predominantly applied uniformly to diversified sites. While select sites in fibronectin domains and DARPins have shown benefit from sitewise designs, they have not been deeply evaluated. Inspired by this disparity between diversity distributions in natural libraries and synthetic scaffold libraries, we hypothesized that binders resulting from discovery and evolution would exhibit a non-spatial, sitewise gradient of amino acid diversity. To identify sitewise diversities consistent with efficient evolution in the context of a hydrophilic fibronectin domain, >10^5^ binders to six targets were evolved and sequenced. Evolutionarily favorable amino acid distributions at 25 sites reveal Shannon entropies (range: 0.3–3.9; median: 2.1; standard deviation: 1.1) supporting the diversity gradient hypothesis. Sitewise constraints in evolved sequences are consistent with complementarity, stability, and consensus biases. Implementation of sitewise constrained diversity enables direct selection of nanomolar affinity binders validating an efficient strategy to balance inter- and intra-molecular interaction demands at each site.

## Introduction

Protein sequence space is immense, and the protein/function landscape is rugged and barren: very similar sequences often have greatly different function with the majority of sequences lacking any utility [1]. Protein complexity and our naivety of sequence/structure/function interplay [2] hinder robust de novo design, although several designs have been successfully realized [3–5]. Thus, naïve identification of protein sequences with novel functions, or even mutants with improved function, benefits from combinatorial analysis of many proteins. The efficacy of this approach is directly dependent on combinatorial library quality and the phenotype selection process. The essence of discovery and evolutionary efficiency is to intelligently search sequence space by identifying the effective extent and amino acid distribution of diversity (if any) at each site. Protein discovery and evolution must balance [6] variance sufficient for generation of novel function (dominated by intermolecular interactions) versus conservation sufficient to maintain a high probability of foldable stability (intramolecular interactions) [7]. This challenge is heightened in small domains [8] that have limited area for a binder interface and require mutation of a larger fraction of the molecule [9].

**Fig 1:**
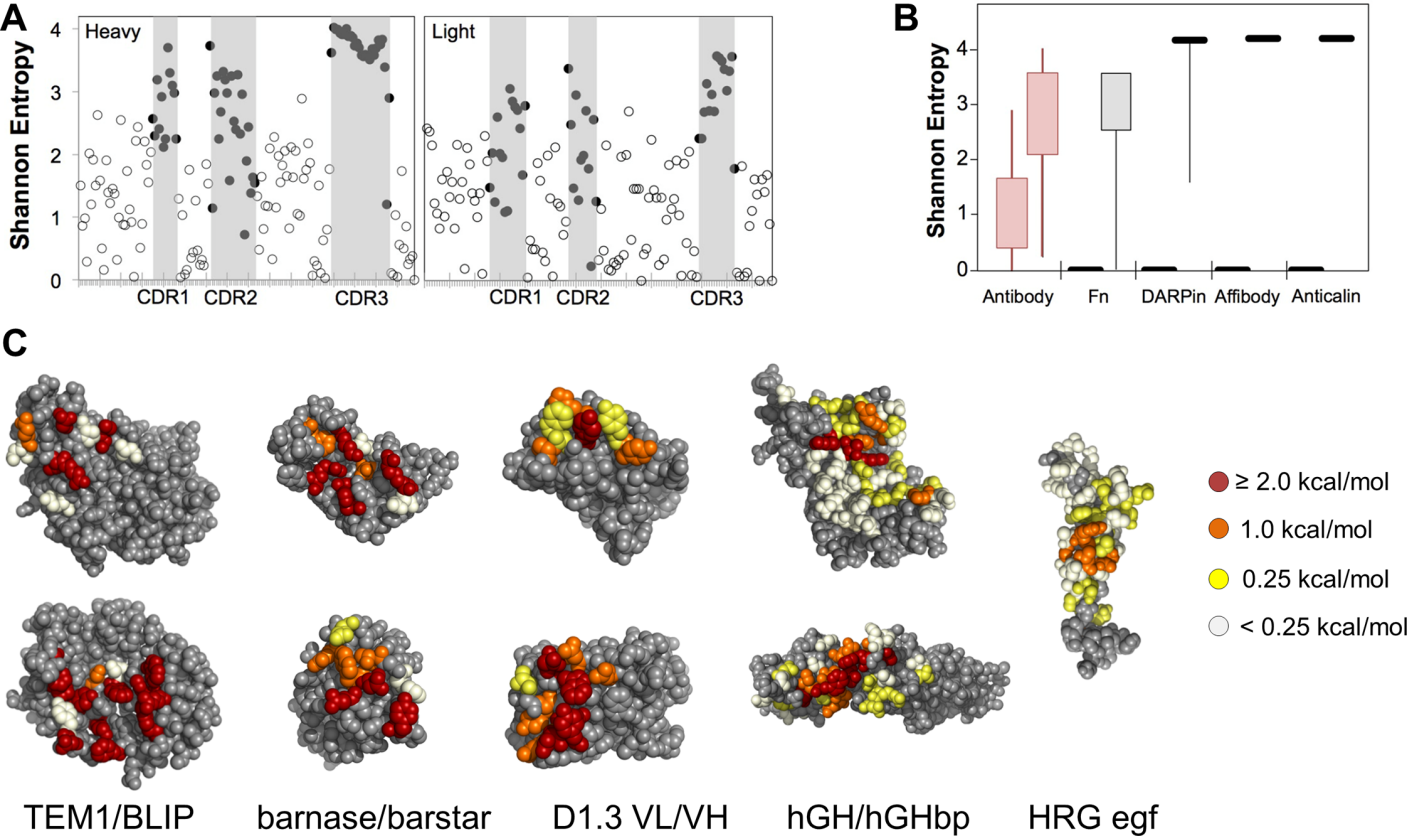
Diversity gradients in binding molecules. (A) The amino acid diversity, measured as Shannon entropy of antibody sequences (from the abysis database at http://www.bioinf.org.uk/abysis/) in the framework (open circles, white background) and CDRs (solid circles, gray background). (B) The Shannon entropy of combinatorial library designs for antibody (data from *A*), fibronectin (Fn) [40], designed ankyrin repeat (DARPin) [41], affibody [42], and anticalin [43], domains. (C) The relative impact of alanine mutation on binding is shown for several protein interfaces: TEM1-β–lactamase (TEM1) and β-lactamase inhibitor protein (BLIP) (PDB: 1JTG) [44]; extracellular RNase (barnase) and its intracellular inhibitory binding partner (barstar) (PDB: 1BRS) [45]; light and heavy chain variable regions of anti-hen egg white lysozyme antibody D1.3 in the context of binding anti-D1.3 antibody E5.2 (not shown) (PDB: 1DVF) [46]; human growth hormone (hGH) and extracellular domain binding partner (hGHpb) (PDB: 1A22) [47,48]; heregulinβ (HRG) egf domain in the context of binding ErbB3 receptor-IgG fusion (not shown) (PDB: 1HAE) [49].

Antibody repertoires have evolved sitewise amino acid distributions across a range of diversities (Fig 1A), which are used in natural and synthetic antibody libraries [10–14]. Yet most synthetic scaffold libraries–including affibodies [15], affitins [16,17], knottins [18,19], anticalins [20], Fynomers [21], Sso7d [22], and OBodies [23]–are binary with a fully conserved framework and uniformly diversified paratope (Fig 1B). Note that different scaffolds use different uniform distributions including NNK codons [24] or complementarity biases [25,26] but generally lack sitewise variation. DARPin domain libraries have six sites with a uniform broad distribution and one site with N/H/Y diversity [27]. A hydrophilic DARPin library includes two additional sitewise diversities [28]. The most sitewise design in non-antibody scaffolds has been introduced in the type III fibronectin domain. Diversification of one, two, or three loops, [29,30] or the sheet surface, [31,32] of this 10 kDa beta sandwich has enabled evolution of binding to a host of molecular targets [30]. Antibody-inspired amino acid bias in putative hot spots has proven effective within fibronectin libraries [31,33–35]. Diversification of two loops is evolutionarily superior to one-loop mutation [36], and although diversification of the third loop (DE loop: G52-T56) is not requisite for high-affinity binding, [30,36–39] it can aid stability [37]. Current library designs randomize G52 with G/S/Y, S53-S55 with Y/S, and 12–22 sites in two other loops with a consistent distribution (30% Y, 15% S, 10% G, 5% each F and W, and 2.5% others except C). Sitewise design was extended beyond the DE loop using accessibility, stability, and homology data yielding nine different diversifications at 11 sites in addition to 12 sites with consistent complementarity-biased diversity [35]. Also, in an alternative paratope approach to engineering fibronectin domains, five sites were identified for three different varieties of constrained diversities (4–8 amino acids) in addition to 12–19 sites with the complementarity biased diversity [31]. While a variety of sitewise diversities have been implemented in the fibronectin domain, the evolved repertoires resulting from these libraries have not been broadly and deeply analyzed.

The current study aims to quantitatively evaluate the broad extents of diversification and sitewise amino acid distributions that evolve in hydrophilic fibronectin domains (Fn3HP) developed as binding ligands. The Fn3HP mutant was previously evolved for hydrophilicity to improve processing and in vivo biodistribution [50]. We posit that a broad repertoire evolved from combinatorial libraries for de novo discovery will exhibit sitewise complementarity-biased amino acid diversity in the binding hot spot [48,51,52], conserved wild-type sequence in the distal framework, and a gradient of diversification at intermediate sites including bias for conservation or interactive neutrality in proximal regions. This gradient is not purely spatial as protein structure and protein-protein interfaces are complex (Fig 1C). Moreover, for novel ligand discovery, the exact paratope is not known ahead of time, which blurs designed localization of a hot spot [51].

The approach used was high-throughput discovery and directed evolution of thousands of binding ligands to various targets from a diverse combinatorial library followed by thorough sequencing of the library and binder populations to identify diversities and amino acids consistent with functional hydrophilic fibronectin domains (S1 Fig). Deep sequencing of evolved protein populations has proven effective for analysis of functionality landscapes for maturation of single protein clones [53], protein families [54,55], and antibody [56] repertoires. Here we apply deep sequencing to several high-throughput discovery and evolutionary campaigns to identify repertoires of evolved hydrophilic fibronectin domains. The results demonstrate a range of diversities and sitewise amino acid preferences consistent with a benefit of a gradient of sitewise constraint. A constrained library based on the observed evolutionary repertoire provides stable, high affinity binders directly without maturation, and the sequence analysis provides a metric to evaluate the balance of inter- and intra-molecular considerations in library design, which are quantitatively assessed.

## Materials and Methods

### Library Construction

Oligonucleotides, including amino acid diversity and loop length variation, were synthesized by IDT DNA Technologies (Tables 1 and 2 and S1 and S2 Tables). Full-length Fn3HP amplicons were assembled by overlap extension PCR. The library of pooled diversified DNA was homologously recombined into a pCT yeast surface display vector [36] within yeast strain EBY100 [57] during electroporation transformation. The protocol was similar to that described by Benatuil et al. [58]. Yeast at an optical density at 600 nM of 1.3–1.5 were washed twice with cold water and once with buffer E (1 M sorbitol, 1 mM CaCl_2_) and resuspended in 0.1 M lithium acetate, 10 mM Tris, 1 mM ethylenediaminetetraacetic acid, pH 7.5. Fresh dithiothreitol was added to 10 mM. Cells were incubated at 30°, 250 rpm for 30 minutes. Cells were washed thrice with cold buffer E and resuspended to 1.4 billion cells per 0.3 mL buffer E. Six μg of linearized pCT vector and 200 pmol of ethanol precipitated gene insert were added and transferred to a 2 mm cuvette. Cells were electroporated at 1.2 kV and 25 μF, diluted in YPD (10 g/L yeast extract, 20 g/L peptone, 20 g/L dextrose), and incubated at 30° for 1 hour. Cells were pelleted and resuspended in 100 mL SD-CAA (16.8 g/L sodium citrate dihydrate, 3.9 g/L citric acid, 20.0 g/L dextrose, 6.7 g/L yeast nitrogen base, 5.0 g/L casamino acids). Plasmid-containing yeast were quantified by dilution plating on SD-CAA agar plates.

**Table 1:** Amino acid diversity encoded in first generation library. Each row was constructed as a separate sublibrary. CDR’ refers to a degenerate codon with the following nucleotide frequencies: 20% A, 15% C, 25% G, and 40% T at site 1, 50% A, 25% C, 15% G, and 10% T at site 2, and 0% A, 45% C, 10% G, and 45% T at site 3. Loop length diversity was afforded at mid-loop positions by inserting CDR’ diversity sites between sites P25-V29 and G79-S85 of the BC and FG loop, respectively, as denoted by subscripts within table. Length diversity at the DE loop occurred between S53-S55, consisting of diversity matching that of K54 as shown below. Note that throughout the manuscript, a series of unseparated capital amino acid abbreviations refer to equally possible amino acids; for example, SYNT indicates 25% each of serine, tyrosine, asparagine, and threonine at that site.

	BC Loop							DE Loop					FG Loop					
Site	D23	A24	P25	AVT 26–28	V29	R30	Y31	G52	S53	K54	S55	T56	T76	GR 77–78	G79	DSPAS 80–84	S85	K86
Sublib. 1	D	A	P	CDR'_1–4_	AST	CDR'	G	G	S	N_0-2_	S	T	T	CDR‘	GDNSYC	CDR’_1–5_	S	N
Sublib. 2	DSYA	AS	PS	CDR'_1–4_	AST	CDR'	GS	GS	S	(NS) _0–2_	S	TS	TS	CDR‘	GDNSYC	CDR‘_1–5_	S	NS
Sublib. 3	DSYA	ASYD	PSYH	CDR'_1–4_	AST	CDR'	GSYCDN	GSYCDN	SYNT	(NSYT) _0–2_	SYNT	TSYN	TSYN	CDR‘	GDNSYC	CDR‘_1–5_	SYNT	NSYT
Sublib. 4	ACDGNSTY	ACDGNSTY	ACDGNSTY	CDR'_1–4_	AST	CDR'	ACDGNSTY	ACDGNSTY	ACDGNSTY	(ACDGNSTY) _0–2_	ACDGNSTY	ACDGNSTY	ACDGNSTY	CDR‘	GDNSYC	CDR‘_1–5_	ACDGNSTY	ACDGNSTY
Sublib. 5	CDR'	CDR'	CDR'	CDR'_1–4_	AST	CDR'	CDR'	CDR'	CDR'	CDR' _0–2_	CDR'	CDR'	CDR'	CDR‘	GDNSYC	CDR‘_1–5_	CDR'	CDR'

**Table 2:** Amino acid diversity encoded in second generation library. CDR’ refers to a degenerate codon with the following nucleotide frequencies: 20% A, 15% C, 25% G, and 40% T at site 1, 50% A, 25% C, 15% G, and 10% T at site 2, and 0% A, 45% C, 10% G, and 45% T at site 3. Loop length diversity was afforded at mid-loop positions by inserting CDR’ diversity sites between sites P25-V29 and G79-S85 of the BC and FG loop, respectively, as denoted by subscripts within table. Length diversity at the DE loop occurred between S53-S55, consisting of either wild-type length or the exclusion of K54.

	BC Loop							DE Loop					FG Loop						
Site	D23	A24	P25	AVT 26–28	V29	R30	Y31	G52	S53	K54	S55	T56	T76	G77	R78	G79	DSPAS 80–84	S85	K86
**Generation 2**	**D**	**A/ASYDNT**	**P/PSYH**	**CDR‘**_**2–4**_	**AST**	**CDR'**	**GY**	**G**	**SYNT**	**(NSYT)**_**0–1**_	**SYNT**	**TSYN**	**TSGA**	**GSYADCNT**	**CDR'**	**GSDN**	**CDR’**_**1–5**_	**S**	**N**

Each resulting Fn3HP naïve yeast library was evaluated for proper library construction by Sanger sequencing clonal plasmids harvested from the transformed yeast (57 clones from generation one and 15 from generation two naïve libraries) and later via Illumina sequencing. The yeast libraries were also labeled with biotinylated anti-HA antibody (goat pAb, Genscript) and anti-c-MYC antibody (9E10, Covance Antibody Products;) to detect the presence of N- and C-terminal epitopes present on either side of the Fn3HP clones, respectively, via flow cytometry. The fractional detection of cells displaying both HA and c-MYC, compared to those displaying HA alone, is indicative of full-length, stop codon-free clones.

### Binder Maturation and Evolution

The Fn3HP yeast library was grown in SD-CAA selection media for several doublings (about 20 h) in an incubator shaker at 30°C until an optical density value of 6.0 was reached, at which time the yeast were centrifuged and resuspended in SG-CAA induction media (10.2 g/L sodium phosphate dibasic heptahydrate, 8.6 g/L sodium phosphate monobasic monohydrate, 19.0 g/L galactose, 1.0 g/L dextrose_,_ 6.7 g/L yeast nitrogen base, 5.0 g/L casamino acids) and grown overnight. The induced library was sorted twice via multivalent magnetic bead selections [59] via depletion of non-specific binders on avidin-coated beads and control protein-coated beads followed by enrichment of specific binders on target-coated beads. The pair of magnetic sorts was followed by a flow cytometry selection for full-length clones using the 9E10 antibody against the C-terminal c-MYC epitope tag. Genes were mutated via error-prone PCR with loop shuffling [37], then electroporated into yeast (EBY100) as previously described. Target binding populations were isolated at two levels of stringency, mid- and high-affinity, for each of the four campaigns. A mid-affinity population included all clones that demonstrated either (i) magnetic bead sorting enrichment at least ten-fold greater for target protein than both avidin binding and non-specific control binding or (ii) binding to 50 nM multivalent target (3:1 stoichiometry of target preloaded on streptavidin-fluorophore) assayed via flow cytometry (S2 Fig). A high-affinity population included all clones exhibiting binding to 50 nM monovalent target assayed via flow cytometry. Herein, clones meeting these criteria are referred to as mid- and high- affinity binders, respectively. Flow cytometry was performed as previously described [60].

### Illumina MiSeq Sample Preparation and Sequence Analysis

Plasmid DNA was isolated from yeast using Zymoprep Yeast Plasmid Miniprep II. DNA samples were divided into separate groups based on library generation of origin and binding affinity. Three categories were included for each generation: naïve clones from the initial libraries, mid-affinity binders collected via magnetic bead sorting, and high-affinity binders collected using FACS. In total, six pools of DNA were isolated and uniquely analyzed in association with generations one and two. Following plasmid DNA extraction, two rounds of PCR were completed to assemble the Fn3HP gene fragment with Illumina primers, index tags, multiplexing bar codes, and TruSeq universal adapter (S4 Table). For all PCR conducted during amplicon library preparation, KAPA HiFi polymerase was used as it has been shown to reduce clonal amplification bias due to GC content [61] as well as fragment length bias [62]. Compatible multiplexing and adapter primers were designed according to TruSeq sample preparation guidelines. Amplicons were pooled and supplemented with 25% PhiX control library to increase MiSeq read accuracy. Illumina MiSeq paired-end sequencing with 2 x 250 read length was conducted (University of Minnesota Genomics Center) to obtain 7.2x10^6^ pass filter (PF) reads from the populations of interest, of which 90% of all pass filter bases were above Q30 quality metric (99.9% read accuracy).

### Sequence Analysis

Raw data generated through MiSeq consisted of forward and reverse read files (FASTQ) for each of the six multiplexed sublibraries. Assembly of paired end reads was done using PANDAseq [63]. Assembled reads were analyzed using in-house Python [64] code. Analysis work flow for each of the six subgroups (e.g. naïve, mid-affinity, high-affinity populations originating from first and second generation libraries) consisted of first identifying full-length fibronectin DNA sequences, isolating each of the three diversified loop regions, and, lastly, calculating the amino acid frequency at each site. Additional calculations were necessary for the mid-affinity and high-affinity populations to both remove statistically rare events and avoid overcounting dominant clones. The removal of background (i.e. the rarest 2% of sequences, as determined by the rarity of nonspecific binders) was a precaution taken when analyzing the mid-affinity populations to account for the rare non-binding clones inherently collected via magnetic bead sorting [59]. To address the potential detriment of overcounting within all binding populations, the sequences for each loop region were clustered based on 80% or greater sequence homology. For each cluster of similar sequences, the summation of the amino acids at each site were weighted by a power of one-half, then aggregated across all clusters. The resulting weighted sitewise amino acid values were used for frequency calculations. Statistical analysis was performed using two sample Student’s t-test. Statistical significance was assessed while adjusting for familywise error rate using Bonferroni method, denoted at level α = 0.005.

### Stability Assessment

High-affinity clones from three separate target binding campaigns of the current study were individually evaluated for stability using thermal denaturation midpoint, T_m_, in the context of yeast surface display, as previously described [37]. Wild-type Fn3HP and seven random clones from the second generation initial library were produced with a C-terminal six-histidine tag in BL21(DE3) and purified by immobilized metal affinity chromatography and reverse phase high performance liquid chromatography. Purified proteins (1 mg/mL in 2 mM 4-(2-Hydroxyethyl)piperazine-1-ethanesulfonic acid, 50 mM NaCl, 2 mM ethylenediaminetetraacetic acid, 1 mM dithiothreitol) were analyzed via circular dichroism [65] using a JASCO J815 instrument. Measurements of molar ellipticity were taken at 218 nm while heating from 20–98°C at a rate of 1°C per minute. Stability measurements of 15 engineered fibronectin clones were retrieved from previously published studies wherein library design was implemented through a binary approach: broadly diversifying the anticipated paratope, using NNS [66] and NNB [37] codons, and fully conserving all other positions.

### Correlative Parametric Analysis of Amino Acid Distributions

To evaluate the correlation of evolved sitewise amino acid frequencies with several computed parameter matrices (sitewise computational stability, sitewise amino acid frequency in natural homologs, complementarity, and exposure), a sitewise amino acid frequency matrix (*F*) is calculated from Eq 1:

$$F=\sum_{k}^{a,b,c}\left(\alpha_{k}+\beta_{k}\;\cdot \;\varepsilon \right)\;\cdot \;f_{k}$$ (Eq. 1)

where α_k_ and β_k_ are tunable weights to scale the primary parameter data (*f*_*k*_) as a function of exposure score, ε (see below). Parameter weights that are most consistent with experimental data were calculated using a least-square method to minimize error between the calculated matrix, *F*, and objective matrix, defined as the sitewise amino acid frequencies observed in binder sequences evolved from the second generation library. Constraints are placed such that each set of α values sum to 1.0 and each set of β values sum to zero.

#### Amino Acid Frequencies in Natural Homologs

The Pfam database offers an extensive collection of protein families compiled through hidden Markov models [67]. The Fn3 protein family homologs (PF00041) were aligned to the 101 amino acids in Fn3HP. To reduce the impact of dominant replicate sequences, while still accounting for their repeated observation, the frequency of replicate sequences were counted as the square root of the total number of occurrences. The amino acid frequency at each site was computed (S3B Table).

#### Stability Matrices

FoldX [68] was used to determine the mutability of sites 23–31, 52–56, and 76–86 within the tenth type III domain of human fibronectin in the context of several structures cataloged in the Protein Data Bank [69] (PDBs: 1FNA, 1TTG, 2OBG, 2OCF, 2QBW, 3CSB, 3CSG, 3K2M, 3QHT, 3RZW, 3UYO). After performing FoldX repair, random mutants were generated for each of the eleven structures by randomizing the BC, DE, and FG loop regions in accordance with the second generation diversity design scheme. At this point, baseline stabilities were individually calculated for each mutant. To analyze the stability impact upon residue substitution for each position in the diversified regions, all 19 natural residue substitutions were individually introduced to the random mutants. The change in stability (ΔΔG_folding_) upon mutation was then calculated for each PDB structure’s collection of mutants. This process was iterated (n > 50) until the ΔΔG_folding_ associated with each position and residue converged to within 0.2 kcal/mol for at least five consecutive mutants. At each site, the stability impact upon substitution to each amino acid was calculated, creating stability matrices for each starting PDB. The sequences corresponding to the wild-type structures were aligned to account for loop length diversity. Average ΔΔG_folding_ values were calculated for all 20 amino acids at each diversified site (S3A Table).

#### Exposure

The likelihood of a loop position to be proximal to or directly involved with a target binding interface is influenced both by exterior exposure of the side chain (i.e. solvent accessible surface area) as well as its proximity to a region offering sufficient diversified surface area to enable the required enthalpic interactions. The site-specific exposure score is calculated as the product of the solvent accessible surface area [35] and an estimated likelihood of residing at the target binding interface. The latter was quantified on a sitewise basis, averaged across eleven Fn3 crystal structures, using a geometric algorithm. Using Python, the fibronectin orientation that presents maximal diversified surface is identified as follows. BC, DE, and FG loop residues are mutated to alanine to remove wild-type residue size bias. The area of accessible (*i*.*e*., visible in a two-dimensional projection as a planar approximation of the interface) diversified surface is calculated for each rotational orientation of fibronectin. The orientation that maximizes accessible view of the paratope, as well as any orientations within 5% of this projected area, is identified starting with a coarse-grained search and optimizing with a fine-grained search. For each site, the maximum area of accessible side chain surface within this set of optimized orientations is calculated. This calculation is repeated for all sites. Sitewise values are averaged across all Fn3 PDB models.

## Results

### Library design and construction

As a collection of starting points for the evolution of diverse ligands, a combinatorial library was created with various levels of diversity throughout the potential paratope of the hydrophilic fibronectin loops (Table 1). Each loop varied in length as guided by natural sequence frequency [37]. The core of the BC and FG loops– 2–5 sites and 2–6 sites, respectively, depending on loop length–had full amino acid diversity biased to mimic the third heavy chain complementarity-determining region (CDR) of antibodies. Two sites spatially within the BC and FG loop cores were constrained based on previous experimental results. V29, which benefits as a small, reasonably hydrophobic amino acid [35,70], was constrained as A, S, or T. G79, which benefits from glycine bias [35], was mildly constrained to G, S, Y, D, N, or C to increase glycine frequency while mimicking CDRs. Twelve sites adjacent to the core of the BC and FG loops were afforded five levels of diversity: i) wild-type, ii) wild-type or serine (as a small, mid-hydrophilicity neutral interactor [71,72]), iii) wild-type, serine, or tyrosine (the most generally effective side chain for complementarity [73]), iv) moderate chemical diversity (A, C, D, G, N, S, T, or Y), or v) full antibody-mimicking amino acid diversity. All framework sites are conserved as the sequence of the tenth type III domain of human fibronectin with the hydrophilic mutations V1S, V4S, V11T, A12N, T16N, L19T, V45S, and V66Q [50] as well as the stabilizing D7N [74]. Five sub-libraries were constructed using separate DNA oligonucleotides with degenerate codons for each level of diversity. The five sub-libraries for each of the three loops were pooled at equimolar levels.

The gene libraries were transformed into a yeast surface display system [57], which yielded 2.0x10^8^ transformants. DNA sequencing of 57 random naïve clones indicated 61% had full-length sequences, 16% contained stop codons naturally arising from the CDR’ diversity, and 21% contained frameshifts. This finding was supported by flow cytometry analysis that revealed 64% of proteins were full-length as evaluated by the presence of a C-terminal c-myc epitope. Thus, the library contained 1.2x10^8^ unique, full-length Fn3HP clones.

### Selection and analysis of binding populations from first generation library

To identify a diverse set of selective ligands for a range of protein epitopes, the pooled library was sorted, using magnetic beads with immobilized protein targets and fluorescence-activated cell sorting (FACS), to yield binders to goat immunoglobulin G (IgG), rabbit IgG, lysozyme, or transferrin. These targets were selected to provide a diverse set of epitopes for targeting. Following a single round of mutagenesis, then two rounds of magnetic bead sorting, an enriched population of mutants was isolated that demonstrated mid-affinity, selective binding to transferrin. Selectivity was evidenced by a 30:1 ratio of fibronectin-displaying yeast selected for binding transferrin relative to binding negative control proteins (avidin and lysozyme). This population was then sorted for high-affinity binders via FACS. Similarly, though with one additional round of mutagenesis, mid- and high-affinity, selective binders for goat IgG, rabbit IgG, and lysozyme were identified (S2 Fig).

The binding populations were sequenced via Illumina MiSeq resulting in 4.2x10^5^ sequences, including 1.1x10^5^ unique sequences. Analysis of similar sequences, identified and clustered using an in-house algorithm, revealed 3,590 unique families of unrelated sequences. Thus, a broad set of evolutionary solutions was identified for Fn3HP-based ligands.

**Fig 2:**
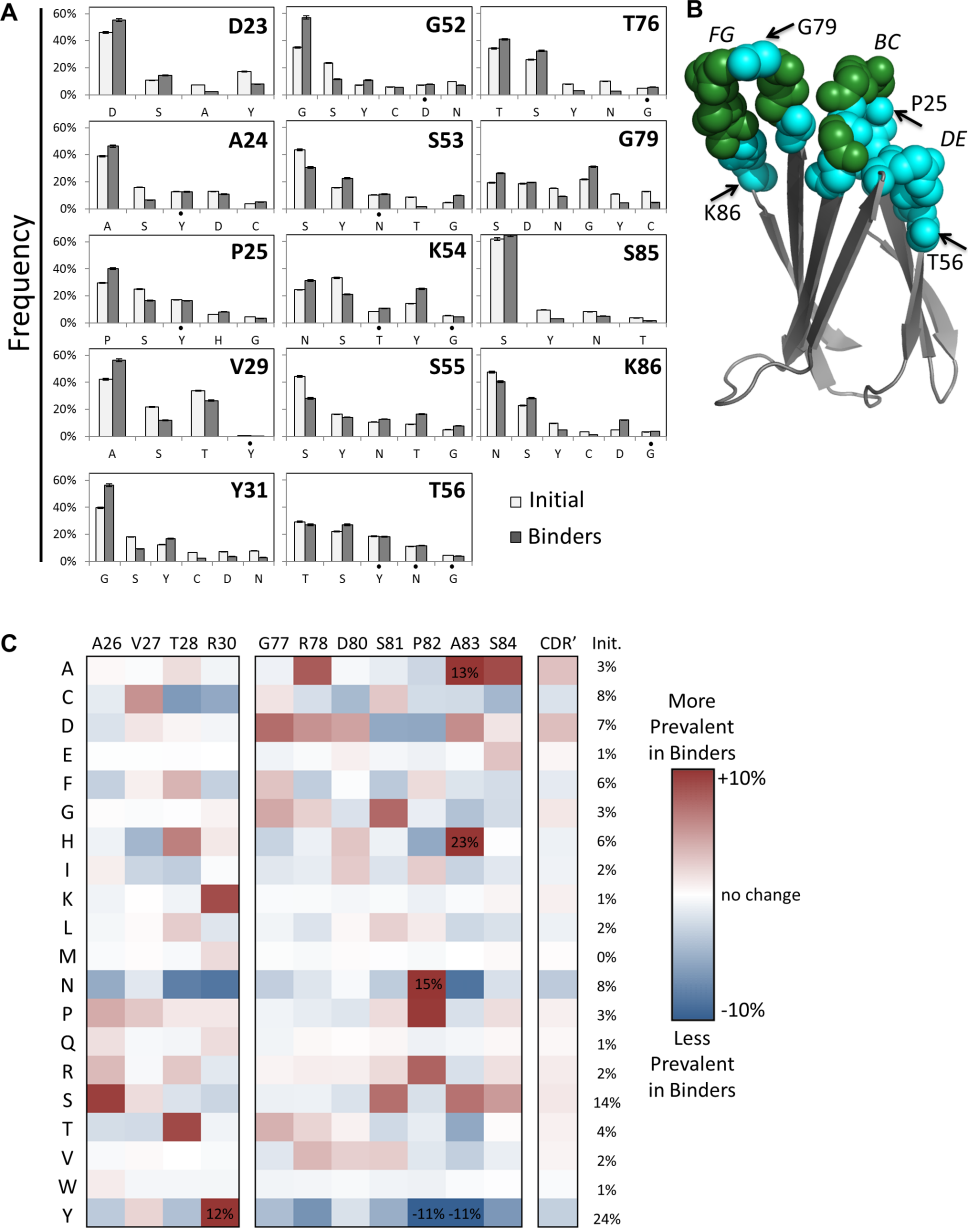
First generation sitewise amino acid distribution. (A) The amino acid frequencies at the indicated sites for the first generation library (white) and the binding populations (gray). Bars and error bars are mean ± standard deviation. Statistical significance, while adjusting for family wise error rate using Bonferroni method, was achieved at level α = 0.005 for all data with the exception of those denoted (•). (B) Solution structure (PDB: 1TTG) of wild-type fibronectin domain with backbone residues of diversified loop sites denoted by spheres. BC, DE, and FG loops are labeled as are several sites for reference. (C) The difference between the amino acid frequencies in the binding populations and the first generation library are shown for each amino acid at each site. The average across all fully diversified sites is also presented. *Init*. indicates the average amino acid frequency in the initial library across all CDR’ sites.

Amino acid frequencies were measured at each site in the naïve and functionally evolved populations to reveal evolutionary impact. The amino acid distribution in evolved ligands exhibits substantial sitewise preferences in both the broadly diversified paratope core (Fig 2C), in which the original library provided complementarity-biased diversity, and the adjacent sites (Fig 2A) in which wild-type and neutral bias were implemented along with lesser complementarity bias. For example, at site 30, N is depleted from 10% in the original library to 1% in evolved clones; conversely, K is enriched from 2% to 11%. At site 85, Y is depleted from 10% to 3% whereas P is enriched from 1% to 12%, suggesting a substantial evolutionary benefit. At site 24, S is depleted from 16% to 6%, and enrichment is broadly distributed by several amino acids. The sitewise amino acid distributions in the population of evolved fibronectin domains, and their deviation from the original unsorted library distributions, can be evaluated in a variety of ways to reveal evolutionary insight.

#### Shannon Entropy

Shannon entropy (H = -∑_i = 1–20_ p_i_ ln p_i_, where p_i_ is the fraction of amino acid i at a particular site), which describes relative diversity ranging from 0 (fully conserved) to 4.3 (5% of each amino acid) [75], was calculated at each site for the naïve and evolved populations to measure constraint within functional ligands. Diversity at 21 of 25 sites is more constrained in the evolved repertoire than in the unsorted library as evidenced by reductions in the Shannon entropies (average: -0.2 units; S3 Fig). Twelve sites have reduction of at least 0.15 units. Notably, G52 (2.8 to 2.0) and G31 (2.7 to 2.1) are largely driven by 22% and 17% increases in constraint of wild-type G. D23 (2.6 to 2.2), N54 (2.8 to 2.4), and T76 (2.8 to 2.4) are driven by broader constraint.

#### Wild-Type Constraint

Even with wild-type constraint averaging 39% in the edges of the BC loop in the original library, wild-type was further enriched in evolved sequences by an average of 11%: D23 (46% to 55%), A24 (39% to 46%), P25 (29% to 40%), and G31 (40% to 56%). Conversely, at the sites in the middle of the loop, fully diversified with complementarity bias, wild-type enrichment was less frequent: absent at A26 (2% to 2%), V27 (<1% to <1%), and R30 (2% to <1%) but present at T28 (6% to 15%).

The FG loop exhibited increases in wild-type constraint throughout: T76, G77, R78, G79, D80, S81, P82, S84, and S85 all elevated wild-type with an average increase of 6%. A83 also increased but with very few sequences available for analysis because of loop length variability. Also, wild-type was not considered at site 86 to eliminate the large, charged side chain K.

In the peripheral DE loop, wild-type constraint was diminished at three of four sites: S53 (44% to 31%), S55 (44% to 28%), and T56 (29% to 27%). Yet at the other site, G52, wild-type was substantially enriched in evolved binders (35% to 57%).

#### Enrichments Predicted by Proteome or Fibronectin Homologs

The extent to which sitewise biases were correlative with wild-type or homologous residues was evaluated. Of 39 residues with an enrichment of at least 5% (in magnitude, not relative increase), 13 are wild-type, 13 are wild-type homologs (A26S, R30K, S55T, T76S, S84A, N86D, N86S, V29A, S53G, S53D, S55D, G79S, and S81G), and 13 are non-homologous as defined by proteome-wide amino acid homology (BLOSUM62 [76]).

Of the 26 residues with enrichment of non-wild-type amino acids, 14 (54%) are also enriched in fibronectin homologs (S3B Table). While these homolog enrichments support the concept of applying consensus design [77] to combinatorial evolution, the other 12 residues (46%) highlight the limitations of such an approach (*i*.*e*., the functional capacity of mutations not observed in natural evolution).

#### Serine

Serine was frequent in the naïve library adjacent to the fully diversified sites because of its purported ability to act as a neutral interactor, providing neither substantial detriment nor benefit. It was mildly reduced (from 29% to 24%) in the evolved repertoire.

#### Sites with Complementarity Bias

On average across all sites with full, complementarity-biased diversity, the biased distribution–based on the distribution observed in natural antibody repertoires–was generally preserved. Modest exceptions were that A and D were enriched by 3% each whereas N and Y were depleted by 3% each.

#### Comparison of Evolutionary Probabilities from the Original and Evolved Repertoires

**Fig 3:**
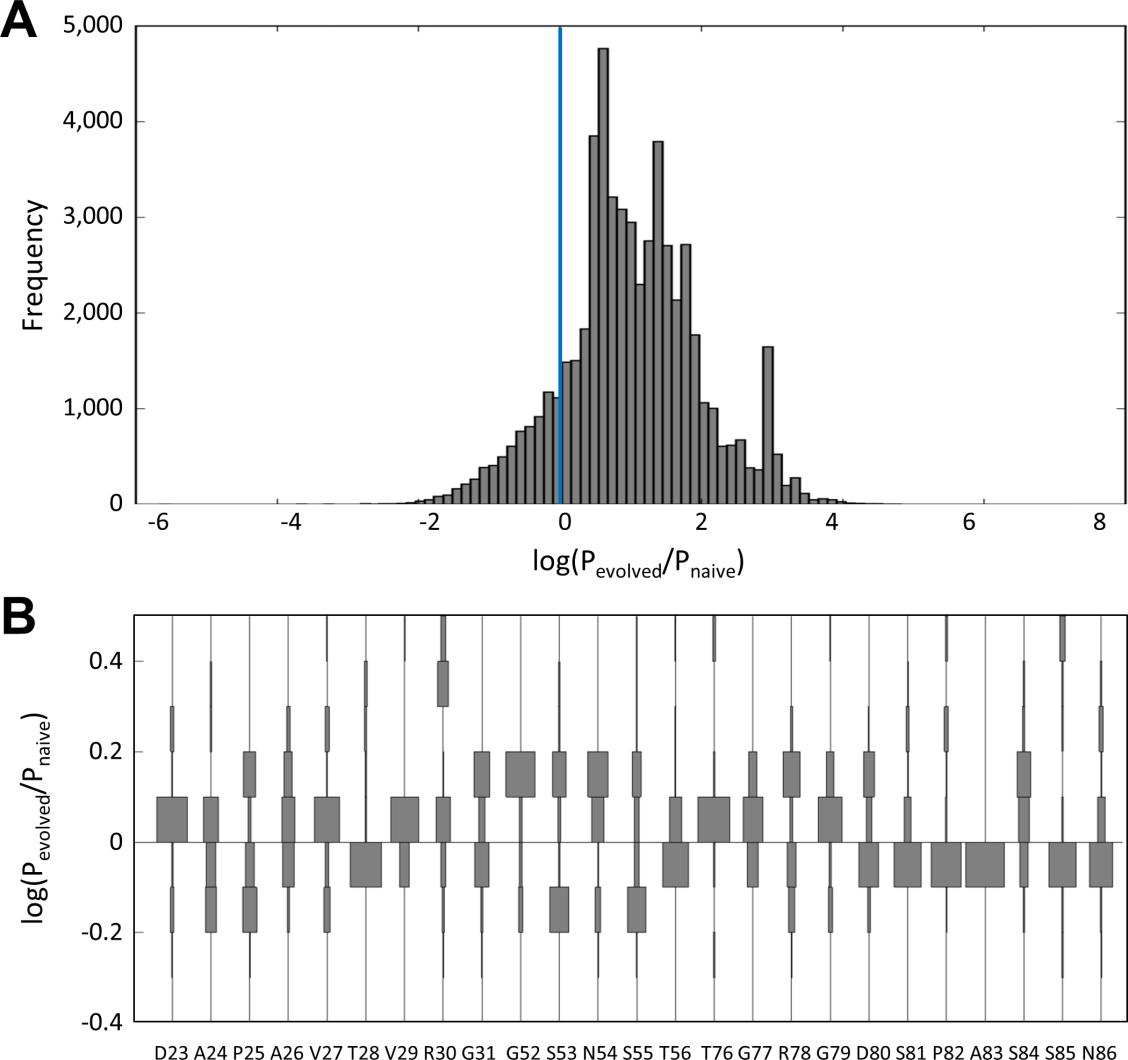
Distribution of evolutionary probabilities from the original and evolved repertoires. To estimate the likelihood of an evolved clone to have been identified from either the first generation repertoire or from that which is observed within the evolved population of binders, an exhaustive leave-one-out cross-validation (LOOCV) was conducted. The training set consisted of n-1 sequence clusters derived from the first generation mid-and high-affinity evolved binder data. Using the training set sequence clusters, a sitewise frequency matrix was calculated. The cross-validation test set consisted of a single sequence cluster that had been excluded from the training set analysis. Each sequence within the test set was assigned a probability for being observed within the original library (P_naive_) and the training set (P_evolved_). Log-odds score, log(P_evolved_/P_naïve_), histograms are shown for test set sequences (A) and further evaluated on a sitewise basis (B).

The original and evolved sitewise amino acid frequencies were compared for their likelihood to yield functional ligands. The repertoire evident in the evolved population is a more efficient starting point for ligand discovery. Using leave-one-out cross-validation–with family clusters partitioned to ensure unbiased training sets (see Materials and Methods for details)– 6.6-fold more clones were more likely to be identified from the constrained repertoire versus the original library (Fig 3A). The median increase in likelihood was 795%; *i*.*e*., an evolved clone was 8-fold more likely to be identified from a combinatorial library based on the new repertoire than the original library. Notably, this original library already had constraint built into it from previous studies, detailed above (particularly constraint to a small hydrophobic residue at 29 and glycine bias at 79).

The probability of evolution from the constrained population versus the original population was also evaluated on a sitewise basis (Fig 3B). Sites exhibit a range of evolutionary enhancements. For example, sites 52 and 54 have 107% and 213% increased likelihood of ligand discovery from the constrained repertoire whereas sites 78 and 81 are essentially neutral (1% and 2% increase towards constrained repertoire).

#### Loop Length Variation

**Fig 4:**
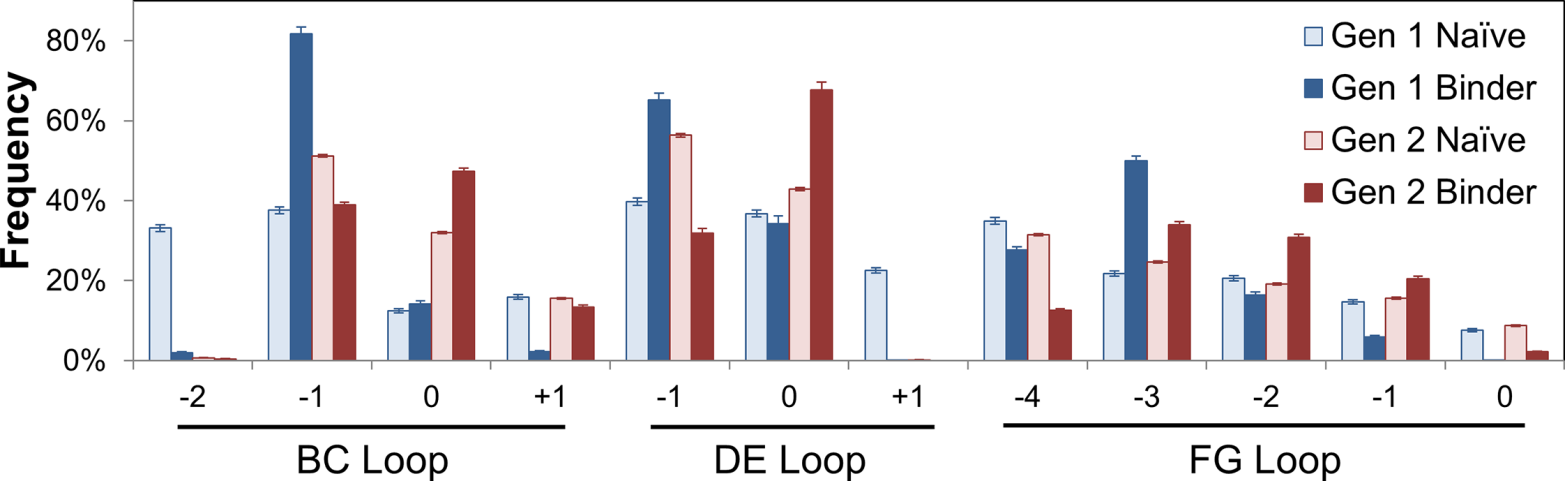
Loop length variation. Loop length frequencies in naïve and binder populations as identified by Illumina sequencing of first and second generation (details in Table 3). Bars and error bars represent mean ± standard deviation.

The BC and DE loops frequently evolved loops one amino acid shorter than wild-type length but also exhibited frequent wild-type lengths (Fig 4). Extended loops were very rare in both BC (2%) and DE (0.1%). Also, a two amino acid deletion in the BC loop, while present in 33% of the original library, only appeared in 2% of evolved domains. Loop lengths were more broadly distributed in the FG loop but with a notable evolutionary preference towards shorter loops.

**Table 3:** High-throughput sequencing. Illumina sequencing statistics for 251 basepair paired end run on MiSeq for six uniquely barcoded libraries. Collectively, 7.2 million pass filter reads were obtained, including 25% PhiX control library. Within all pass filter read bases, 90% were above Q30 quality metric (99.9% read accuracy). Thorough sampling was observed across all six libraries with a coefficient of variance of 30%.

	Generation 1		Generation 2	
	Bead	Flow	Bead	Flow
Total Sequences	181,716	239,934	259,519	225,205
Unique Sequences	65,971	47,957	128,485	103,615
Unique Families	3,334	256	3,733	1,234

### Design, construction, selection, and analysis of binding populations from second generation library

#### Library Design Based on First Generation Evolved Repertoire

The amino acid distributions observed in binding ligands evolved from the first generation library were used to design a second generation library, which enables further study of evolutionary repertoires from a more constrained library as well as evolution of useful ligands. Within the BC loop at site 23, wild-type D was enriched (46% in the naïve library to 55% in binders) whereas Y, introduced at 17% because of complementarity bias, was substantially depleted in binders to 8±1%. Small, mildly hydrophilic residues were also enriched–G (5% to 11%) and S (11% to 14%)–but not to levels comparable with wild-type bias. Thus, D23 was conserved in the second generation library. At site 24, wild-type A was elevated (39% to 46%), Y was maintained (13% to 13%), and S was substantially depleted (16% to 6%). Thus, the options of wild-type conservation and mild diversity were further explored in the second generation. At site 25, wild-type P was enriched (29% to 40%), Y was maintained (17% to 16%), S slightly declined (25% to 17%), and H was enriched (6% to 8%). Wild-type conservation appears beneficial yet Y, S, and H warrant further consideration. At site 29, the more hydrophobic A was enriched (42% to 56%) whereas the more hydrophilic S and T were depleted (22% to 12% and 34% to 26%, respectively) but still frequent. Thus, second generation design maintained a distribution of AST. Y31 was enriched from 12% to 17% in binders. Glycine, which occurs with 31% frequency at this site within natural sequences of homologous proteins, increased in prevalence from 40% to 56%. Substantial decreases in S (18% to 9%), N (8% to 3%), and C (7% to 2%) were observed. The second generation library contained GY diversity. Wild-type G52 was enriched (35% to 57%) whereas S was depleted (23% to 11%). Wild-type conservation appears strongly beneficial at site 52. At sites 53–55, Y and N were enriched or maintained whereas S was depleted, but still present at reasonable levels. Thus, the second generation library implemented YNST diversity at these sites. T56 exhibited similar results without S depletion leading to TYSN design. At FG loop site 76, enrichment was observed for both wild-type T (34% to 41%) and S (26% to 32%) whereas N (10% to 3%) and Y (8% to 3%) were depleted in binders. Thus, the next design included T and S as well as the other small mid-hydrophilic G and A. At site 79, wild-type G was enriched (22% to 31%) as was S (19% to 27%). D (19% to 20%) was maintained but C (13% to 5%), Y (11% to 5%), and N (15% to 9%) decreased. Thus, GSDN diversity was used in the second library. Wild-type S85 maintained (62% to 65%), which prompts future conservation. At site 86, N was mildly decreased (48% to 40%) while S increased from 23% to 28% and Y decreased from 10% to 5%. The second generation design was intended to be NS but was erroneously synthesized as conserved N.

At G77, wild-type was enriched from 5% to 9% in binders. Y remained frequent in binders (20% to 16%), and D (11% to 18%) and T (4% to 8%) were enriched. Thus, G77 was set to GSYADTNC in generation two. Though several enrichments and depletions are evident elsewhere, all other CDR’ sites will be maintained as CDR’.

The second generation library (Table 2) was constructed from degenerate oligonucleotides. 4.2x10^9^ yeast transformants were obtained. 71% were full-length as assessed by cytometry and corroborated by Sanger sequencing (67% full-length).

#### Selection and Analysis

**Fig 5:**
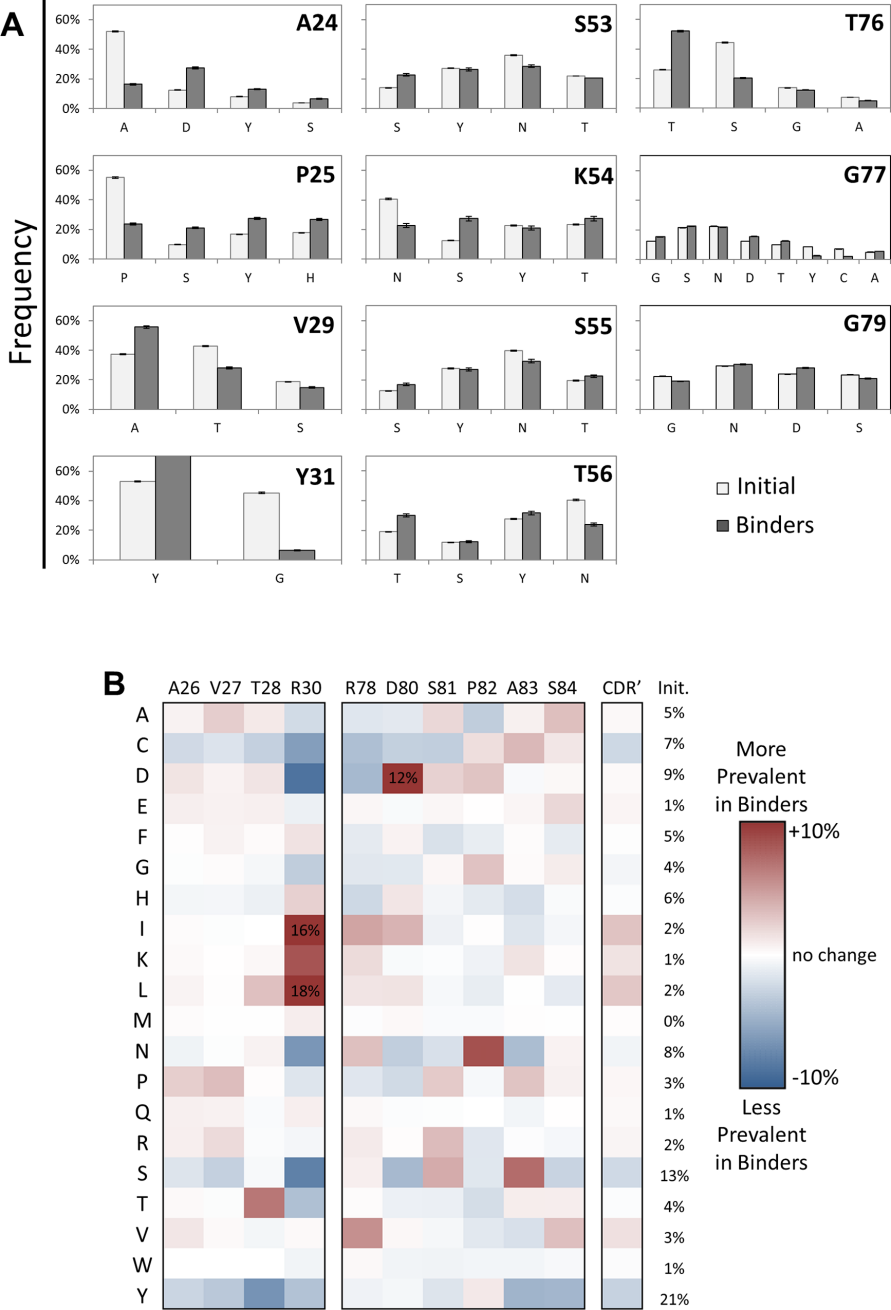
Second generation sitewise amino acid distribution. (A) The amino acid frequencies at the indicated sites for the second generation library (white) and the binding populations (gray). Bars and error bars are mean ± standard deviation. (B) The difference between the amino acid frequencies in the binding population and the second generation library are shown for each amino acid at each site. The average across all fully diversified sites is also presented. *Init*. indicates the average amino acid frequency in the initial library across all CDR’ sites.

Mid- and high-affinity binders to MET, lysozyme, and rabbit IgG, as well as mid-affinity binders for tumor necrosis factor receptor superfamily member 10b, were evolved and sequenced using Illumina MiSeq with barcodes to identify mid- and high-affinity binders. Sequences were aligned, clustered, and counted, with accommodations to reduce overcounting of highly similar sequences, using an in-house algorithm. 4.8x10^5^ sequences were collected with 2.3x10^5^ identified as unique (Table 3). The sitewise differences between amino acid frequencies in the naïve library and selected binders were calculated at uniquely constrained sites (Fig 5A) and CDR’ sites (Fig 5B).

Sites 24 and 25 exhibit similar results in which significant wild-type conservation was not maintained in binders (52% to 16% of A24 and 55% to 24% of P25) and the other amino acid options were elevated fairly uniformly. At site 29, alanine was increased (37% to 56%) while threonine was depleted (43% to 28%). At site 31, wild-type tyrosine is substantially enriched (53% to 92%) at the expense of glycine (45% to 7%).

In the middle of the DE loop, sites 53–55, asparagines were depleted from their overly high starting points (36% to 29%, 41% to 23%, and 40% to 33%) while serines, which were more rare than designed in the naïve library, were increased (14 to 23%, 13% to 27%, and 13 to 17%). Y and T were essentially maintained thereby supporting the SYNT diversity when equally implemented. Asparagine was also decreased at site 56 (41% to 24%), but wild-type threonine was preferentially increased (19% to 30%).

At the edge of the FG loop, wild-type T76 was increased from 26% to 52% in binders while serine was decreased from 44% to 20%. At site 77, wild-type G (12% to 15%) and aspartic acid (12% to 16%) were enriched, serine (21% to 22%) and asparagine (22% to 22%) were maintained, and tyrosine (9% to 3%) and cysteine (7% to 2%) were depleted. At site 79, the GDSN diversity was consistently maintained in binders.

#### Sites with Complementarity Bias

**Fig 6:**
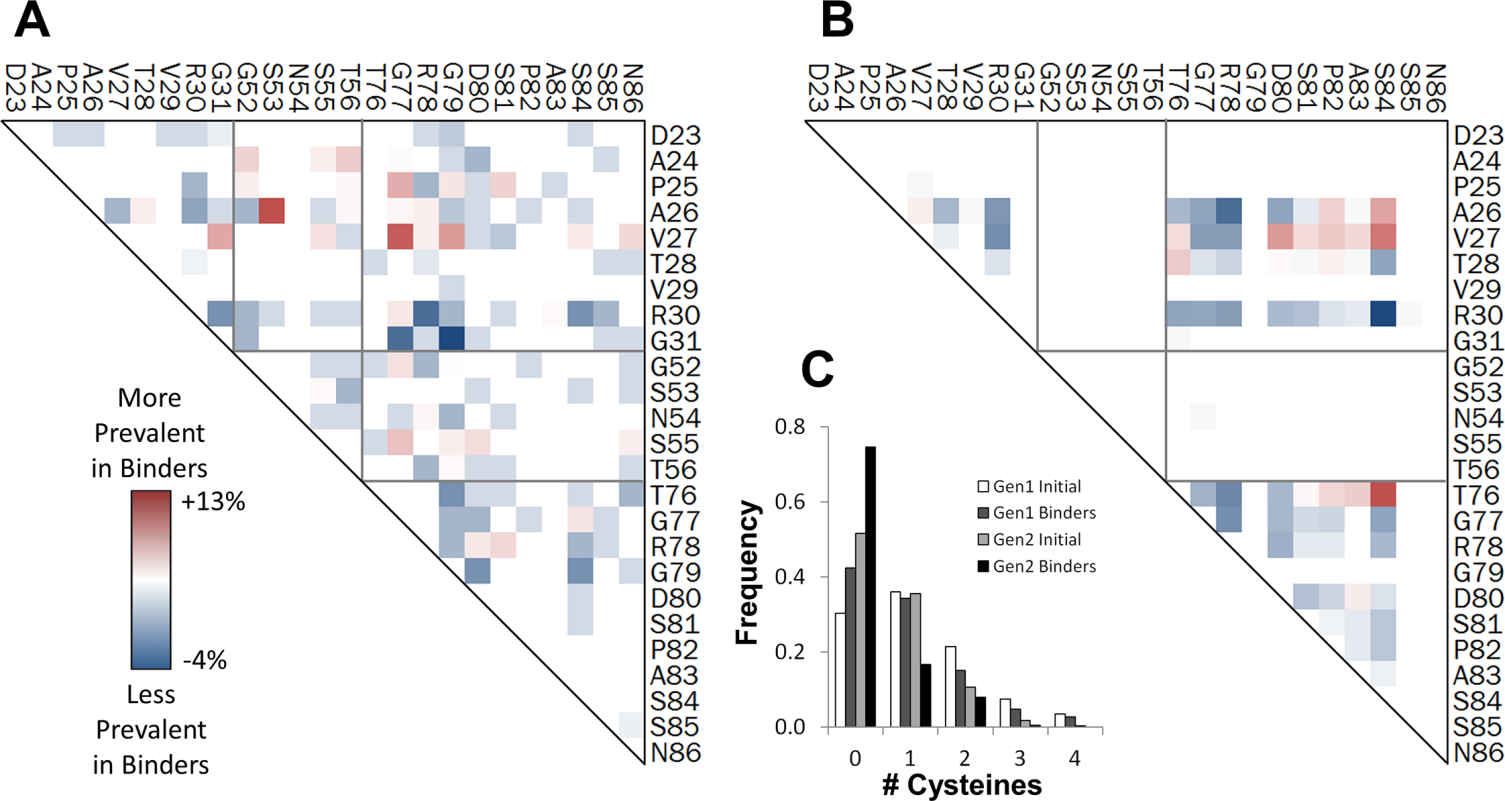
Cysteine frequency analysis. (A, B) Change in pairwise frequency of clones containing exactly two cysteines (high-affinity populations minus naïve library) for the first generation (A) and second generation (B) libraries. (C) Frequencies of clones containing the indicated number of cysteines in initial libraries and binder populations.

In the fully diversified sites, the antibody-inspired diversity was maintained for many amino acids. Sitewise exceptions include wild-type conservation at D80 (9% to 22%), T28 (4% to 11%), and S81 (14% to 18%) and enrichment of isoleucine and leucine at site 30 (4% to 38%). Slight overall exceptions–decrease in cysteine (7% to 4%) and increases in hydrophobics isoleucine, leucine, and valine (sum 8% to 15%)–all compensate for imperfections in the degenerate codons, yielding frequencies more in line with natural antibody repertoires. The decrease of cysteine residues is driven by a lack of enrichment of single-cysteine clones more than depletion of dual-cysteine clones, which is perhaps suggestive of beneficial disulfide bond formation (Fig 6). Evaluation of cysteine pairs in dual-cysteine clones indicates a strong enrichment of clones with a cysteine in sites 26–28, especially 27, of the BC loop and 80–84 of the FG loop. Simultaneous cysteines at sites 76 and 84 are also frequently selected in binders.

#### Loop Length Variation

Loop length analysis indicated diverse lengths were used in binders with a preference for wild-type lengths, or one amino acid decreases, in the BC and DE loops and a broader distribution in the FG loop with preference for 1–3 residues less than wild-type (Fig 4). The longest FG loop, which is only observed in the tenth type III domain of human fibronectin but not the other fourteen human type III domains, is rarely observed (2%) in binders. The shortest FG loop was also less frequently observed in binders (13%) than in the unsorted library (31%).

#### Framework Mutations

The framework sites that were intended for conservation were also analyzed within the naïve and binder sequences to identify mutations, occurring during oligonucleotide synthesis, gene assembly, or directed evolution, that were preferentially present in binding clones. Four mutations were enriched (Table 4). Notably, the P44S mutation, and to a lesser extent S43F, are likely introduced by polyadenylation of the 3’ tail by Taq polymerase during error-prone PCR [78] and then amplified by evolutionary selection.

**Table 4:** Enrichment of framework mutations. Full length fibronectin sequences from first and second generation naïve and binder populations were analyzed. Four framework positions demonstrated enrichment for non-wild type residues. Prevalence of these amino acids in natural homologs is shown in the two right most columns. Bottom row indicates median values of wild-type and any single mutant across all sites.

	Frequency in this work				Natural Frequency	
Mutation	First Generation		Second Generation		Wild-type	Mutant
	Initial	Binders	Initial	Binders		
I20V	0.5%	8%	0.5%	2%	16%	30%
S43F	0.6%	10%	1%	12%	19%	1%
P44S	17%	27%	11%	34%	23%	7%
I88T	0.1%	1%	0.2%	6%	13%	3%
median (all sites)	<0.01%	<0.01%	<0.01%	0.01%	21%	2%

#### Diversity

**Fig 7:**
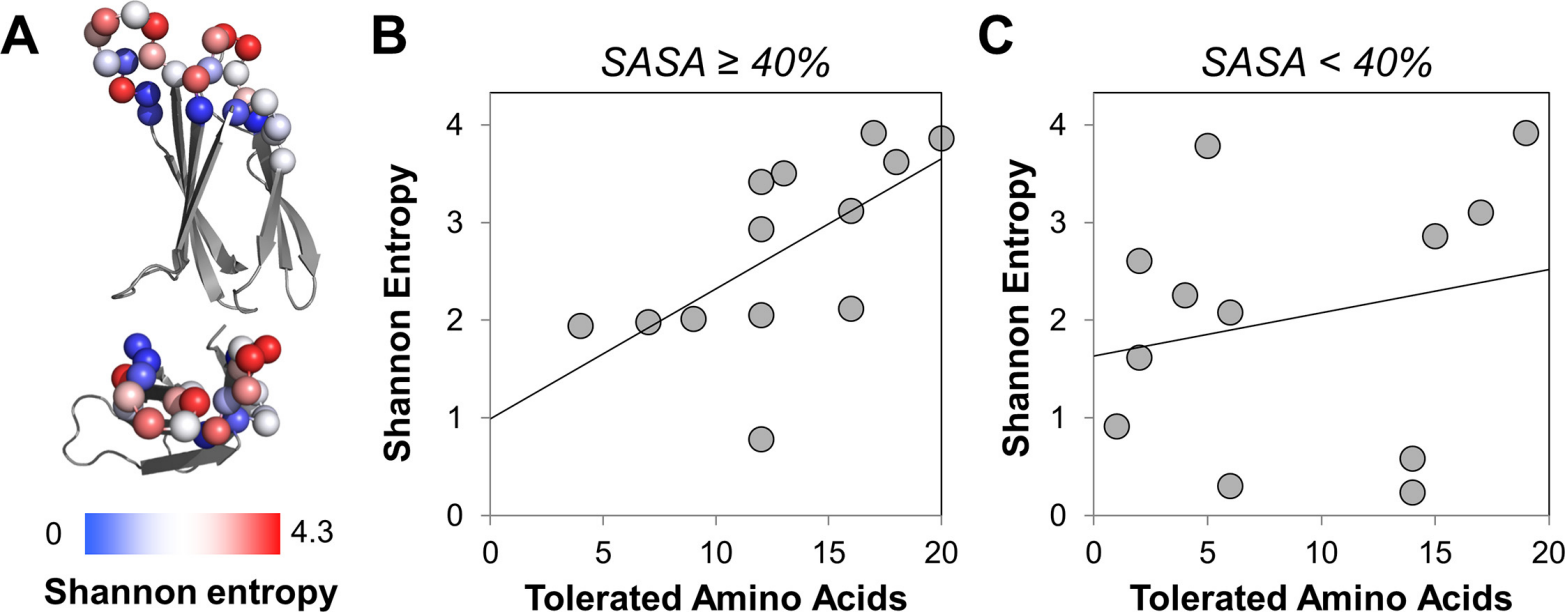
Shannon entropy landscape among binding population. (A) The α carbons of evaluated sites are shown in spheres colored based on Shannon entropy of binding sequences from the second generation campaigns. (B, C) The Shannon entropies at each site of second generation binders are plotted versus the number of amino acids that are tolerated at that site based on computational stability predictions. (B) Sites with solvent accessible surface area (in other fibronectin domain mutants) ≥40%. Pearson coefficient = 0.63. Slope = 0.13. (C) Sites with solvent accessible surface area <40%. Pearson coefficient = 0.22. Slope = 0.04.

The binders generated from the second generation library exhibit a range of sitewise amino acid frequency distributions that are not purely spatial (Fig 7A). Four sites exhibit Shannon entropies [75,79] in excess of 3.5. Three additional sites are in excess of 3.0. Nine sites have entropies from 2.0–3.0. Six diversified sites exhibit Shannon entropies below 2.0. Four sites were conserved, as designed, based on first generation library analysis.

#### Binder phenotypic characterization

**Fig 8:**
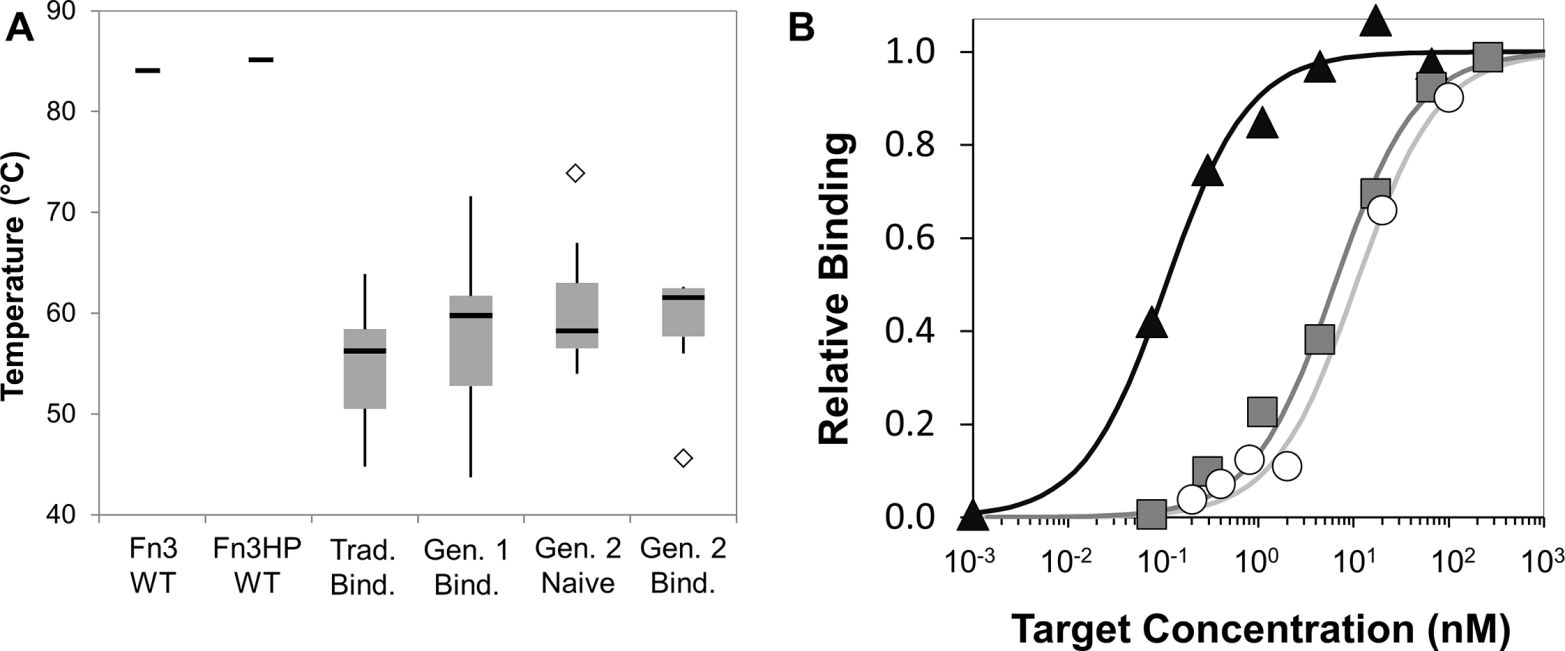
Clonal characterizations. (A) Thermal stabilities of wild-type (WT) fibronectin [80] and Fn3HP are shown. Median (black line), second and third quartiles (gray box), upper and lower inner fences (vertical lines), and outliers (diamonds) are shown for a sampling of clones from binarily diversified traditional libraries [37,66,80] (n = 15, *Trad*. *Bind*.) and three populations of the current study: first generation library binders, second generation naïve library, and second generation library binders. (B) Affinity titrations. Yeast displaying Fn3HP variants were incubated with the indicated concentration of biotinylated target molecule. Binding was detected by streptavidin-fluorophore and flow cytometry. Data points are from a single representative experiment. Affinities were calculated as 150 ±60 pM (rabbit IgG, clone 0.6.2, black triangles), 4 ±3 nM (lysozyme, clone 0.6.3, gray squares), and 11 nM (MET, clone 3.4.3, white circles).

By constraining diversity at select sites, we aim to improve the balance of inter- and intra-molecular interaction evolution and reduce destabilization upon mutation. Thus, we evaluated the stability of several fibronectin mutant populations: binders from both library generations in this work and binders evolved from binary (fully conserved framework, fully diversified loops) libraries from previous literature as well as the naïve second generation population and the parental fibronectin domains (human and hydrophilic mutant) (Fig 8A). The first, second, and third quartile stabilities are higher for the first and second generation libraries relative to binders from less biased libraries. Further still, the median stability of the less biased library binders is less than even that of the naïve members of the second generation library (p < 0.05). Note that Fn3HP is of essentially equivalent stability as Fn3 (84°C vs. 85°C).

In addition to yielding stable binders, the second generation library yields high-affinity binders with little to no evolution. Three binder campaigns continued with additional sorts to identify the strongest binders in the population. Rabbit IgG and lysozyme binders were characterized following two rounds of magnetic bead selection, one round of cytometry sorting at target concentrations of 50 nM and a final round of cytometry sorting at 1 nM, wherein the top 1% of binding events were isolated. Titrations curves of representative clones from the most stringently sorted, non-evolved rabbit IgG and lysozyme populations (Fig 8B) revealed affinities of 150±60 pM and 4±3 nM, respectively. High-affinity MET binders were isolated following three iterations of evolution, each iteration consisting of two magnetic bead selections, one cytometry sort for full-length clones, and one round of error-prone PCR. A representative clone from the evolved MET binding population yielded an affinity of 11 nM (Fig 8B).

### Library Design Principles

The high-throughput binder engineering and analysis described herein provides one means of identifying the extents of diversification, as well as the relevant amino acids, at each site. To further explore the broad sequence data set of highly functional clones resulting from this study, we examined if any computational means could have guided this library refinement; *i*.*e*. if any computable parameters correlate with the evolved repertoire. The FoldX algorithm [68,81] was used to predict each site’s tolerance to mutation. The change in stability (∆∆G_folding_) upon mutation across >500 theoretical library variants (see Materials and Methods) was predicted for each of the twenty amino acids at each site. The mutational tolerance was assessed in terms of the number of minimally destabilizing (∆∆G_folding_ < 0.75 kcal/mol) amino acid substitutions allowed at each site. Observed amino acid diversity (Shannon entropy) in evolved binders correlated with computational mutational tolerance at exposed sites (Fig 7B). While no correlation was observed for less exposed sites (Fig 7C), it should be noted that the key outliers are the sites most distant from the paratope center (D23 and S85).

More broadly, four elements were evaluated for their correlation with evolved repertoires: sitewise amino acid frequencies from natural fibronectin homologs, sitewise computational stabilities of each amino acid at each site within the context of diverse fibronectin clones, complementarity-biased amino acid distributions observed in antibody CDRs, and sitewise sidechain exposure. The first two elements–frequency in natural homologs and computational stability–provide sitewise amino acid distributions; complementarity bias provides a single site-independent amino acid distribution; and the fourth element–residue exposure–provides a sitewise weight. We examined the ability of these four elements to combine to generate sitewise amino acid distributions that matched the experimentally observed frequencies. The relative weights of the first three elements were allowed to vary for each site based purely on the fourth element: solvent and target accessibility of that site (Eq 1). Sitewise frequencies in natural homologs were calculated from 58,058 homologs from the Pfam database [82]. Sitewise computational stabilities were calculated using FoldX as described above. Complementarity bias was calculated as the amino acid distribution observed in expressed human and mouse antibody CDR-H3 sequences [83]. Solvent accessibility is the relative solvent accessible surface area [84] of each side chain averaged over 11 fibronectin structures. Target accessibility was scored based on the orientation of the amino acid side chain relative to the rest of the diversified paratope (detailed in Materials and Methods).

**Fig 9:**
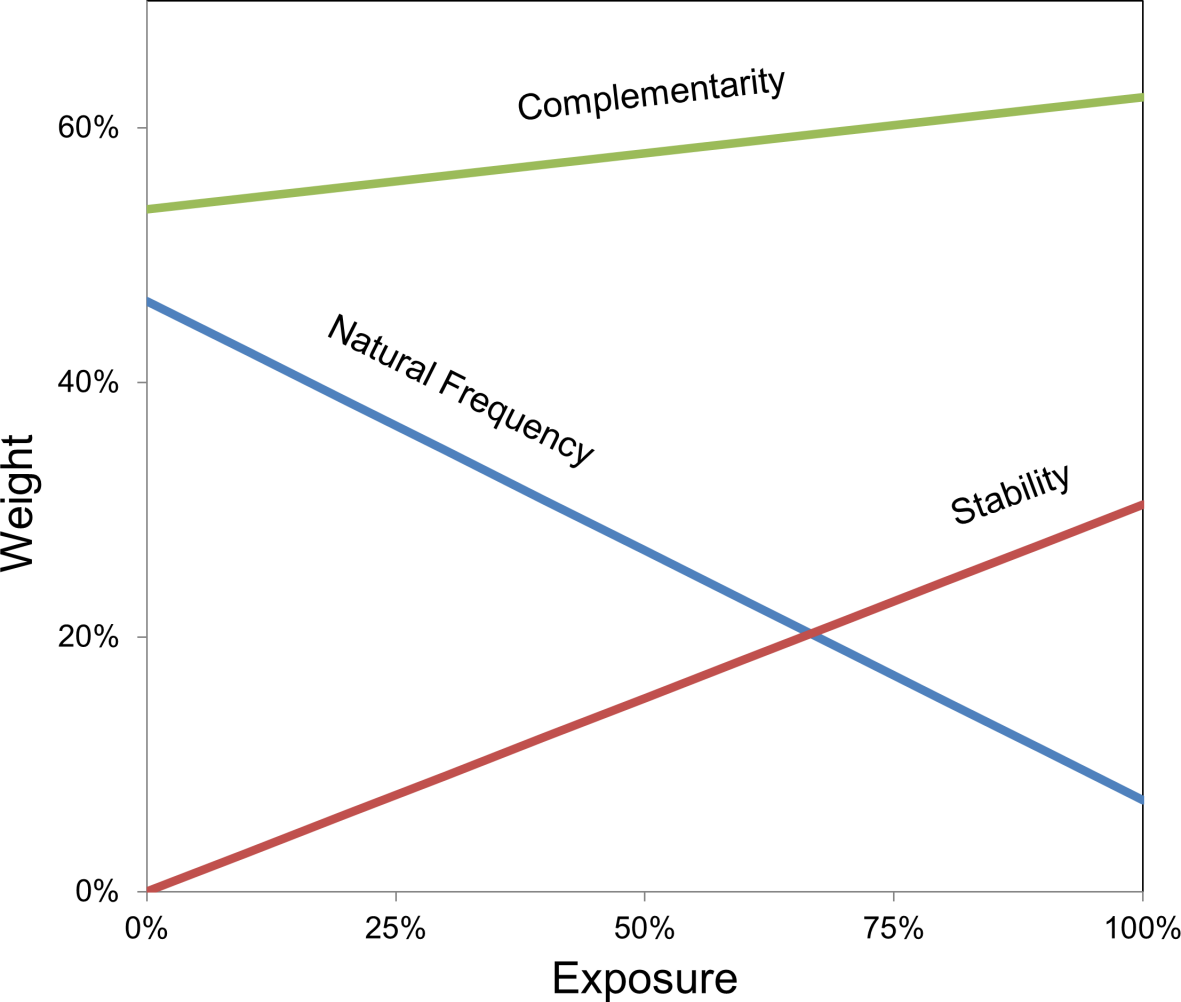
Correlative parametric analysis of amino acid distributions. Sitewise evaluation of theoretical stability upon mutation and natural sequence frequency, as well as overall amino acid prevalence at binding interfaces of antibodies (i.e. complementarity), generate sitewise amino acid frequencies. The ability of these frequencies–scaled linearly based on solvent exposure and target exposure (Eq 1 and S3 Fig)–to collectively mimic the observed sitewise amino acid distributions in binding populations is evaluated. The optimal weights for each contributing data set as a function of exposure are shown.

The relative weights of homolog frequency, computational stability, and complementarity bias were computed that, when linearly combined, yield a sitewise amino acid distribution that is most consistent with the evolved distribution. Weights were calculated for both an exposure-dependent and exposure-independent term, which were summed. The weights provide a relative comparison of the predictive value of each parameter. To evaluate the predictive value overall, the parameters were compared to an unbiased control input matrix (uniformly 5% of each amino acid). Weighted inclusion of all elements yields a library design that matches the experimentally evolved distribution 22 standard deviations superior to designs based on unbiased input matrices (S3 Fig). For well-exposed sites, amino acid diversity is effectively mimicked by 62% complementarity bias, 30% stability computation, and 8% natural frequency (Fig 9). At sites with less exposure, natural amino acid frequency should be more heavily weighted at the expense of stability and, less so, complementarity. Design based on a single element is inferior to randomness for stability, marginally effective (2 standard deviations above random) for natural frequency, and strongly effective (16 standard deviations above random) for complementarity. In short, the evolved repertoire correlates strongly with complementarity and moderately with computed stability and frequency in natural homologs, varying dependent upon sidechain exposure.

## Discussion

In the pursuit of a broadly functional combinatorial library capable of yielding binders to numerous targets, the benefit of diversification is unclear for sites peripheral [52,85,86] to a ‘hot spot’ that enthalpically drives high-affinity binding [51,52,87]. Moreover, the location of the hot spot can differ across different epitopes being targeted by a scaffold. These peripheral sites can (a) directly contact target, (b,c) impact neighboring residue orientation to improve interfacial enthalpy or reduce entropic penalty upon binding, and/or (d) stabilize the protein. Yet these potential benefits can be offset by the inverse impacts: make unfavorable interfacial contact, worsen neighboring residue orientation, and/or destabilize the protein. If sufficient ‘hot spot’ interfacial area is not yet present for high-affinity binding, then additional sites must be diversified to enable favorable interaction. At some point, this expanded paratope provides sufficient interface for strong, specific affinity. Similar tradeoffs can be considered for peripheral sites. Given the typical detriment of random mutation, [88–90] the average peripheral mutation will hinder all four elements thereby suggesting against diversity. Though as a corollary, on average, mutations in the ‘hot spot’ will negatively impact the last two elements by worsening the entropic penalty upon binding and destabilizing the protein because of imperfect interactions with the conserved peripherals. Thus, peripherals need to be chosen to make neutral to good contact with: (a) intermolecular target; (b,c) intramolecular neighbors involved in binding; and (d) all intramolecular neighbors. For *a-c*, since beneficial interactions will be unlikely, amino acids–such as serine [25,72,91]–that yield relatively neutral interactions may be wise. For *d*, beneficial interactions are likely for the wild-type residue and conserved neighbors based on their coevolution so conservation should be the aim. Since the precise locations of the hot spots and these transitions will vary for each new ligand-target interface (Fig 1C), we hypothesized the evolved repertoires will exhibit a gradient of diversity from extensive diversity in the potential paratope hot spot to full conservation in the framework. Importantly, this gradient includes moderate diversity, with structural bias, within the paratope interfacing with target yet peripheral to the hot spot. Moreover, more mild diversity is included adjacent to the interfacial residues to yield optimal intramolecular contacts with the newly identified paratope. The range of Shannon entropies (Fig 7) and amino acid frequency distributions (Figs 2 and 5) across many binding sequences against several targets support the benefit of a gradient of diversities within combinatorial libraries. The particular amino acid distributions support the hypothesized benefits of wild-type conservation, serine bias, and complementarity bias, at appropriate sites.

Sitewise optimization of this gradient between intra- and inter-molecular interaction biases can be achieved with broad, high-throughput binder generation and deep sequencing as demonstrated here. Yet this requires a sufficiently effective library to generate numerous binders, which may be difficult for new scaffolds or paratopes. Analysis conducted in the current study (Fig 9) provides further evidence that initial combinatorial library design can be guided by complementarity-determining residues and, when available, natural homolog frequencies, stability data (theoretical or experimental), and side chain exposure to solvent and target. Ongoing studies can quantify the values of complementarity, stability, natural sequence frequency, exposure–and potentially other metrics–in the context of other protein topologies and other functions, such as catalysis.

Sitewise optimization of amino acid frequency, with a range of diversities, can be implemented in numerous ways. Trimer phosphoramidite codons can be used in oligonucleotide synthesis, [14] which enables precise control over each distribution but elevates synthesis complexity and cost. Independent oligonucleotides can be synthesized for each loop sequence, which further elevates control by enabling pairwise (and higher order) site design albeit at an elevated synthesis scope. Simpler, less expensive single-nucleotide mixed degenerate oligonucleotide synthesis can approximate many amino acid distributions, especially with the inclusion of unbalanced nucleotide frequencies as used in this study. The amino acid distribution within antibody CDR-H3 can be closely approximated by unbalanced single-nucleotide methods [35], but it must compromise on the genetic code connectivity of glycine, tyrosine, and cysteine. Achieving the desired high frequencies of tyrosine (20%) and glycine (16%) yields much more cysteine than desired (10%). In these libraries, we opted to maintain high tyrosine (17%) while limiting cysteine (5%) at the expense of low glycine (4%). Wild-type glycine bias at sites G52 and G79, as well as G77 in the second generation library, enabled this successful compromise as glycine at fully diversified sites was only marginally enriched in binders relative to the original library (2.7% to 4.0% in the first generation; 3.8% to 4.7% in the second generation). Thus, selective sitewise bias is able to effectively provide the evolutionary benefit of the presence of glycine within the DE and FG loops.

Note that the sublibrary synthesis approach in generation one (Table 1) yields coupling between sites within each loop. For example, wild-type D23 conservation pulls wild-type conservation in other BC sites during generation one analysis. In the absence of this coupling in generation two analysis, wild-type conservation at other BC sites (A24, P25, and Y31) is reduced. In the DE loop, wild-type G52 conservation pulls N54 conservation, which converts to N54 depletion in generation two in the absence of G52 coupling. Thus, when evaluating a new scaffold or paratope design, sublibrary construction enables analysis of numerous diversification strategies, but care must be taken to consider coupled sites.

While cysteines were overall depleted from binding sequences relative to the naïve library, select inter- and intra-loop cysteine pairs were enriched. These occurred at proximal locations that are structurally sensible for disulfide bond formation, but further validation is needed to confirm the existence of disulfide bonding. Enhanced evolutionary efficiency of this class of clones warrants consideration of biased design to drive the conformational restriction beneficial to numerous topologies including stapled helical peptides [92], shark new antigen receptors [93], camelid antibody domains [94], and previous fibronectin clones [36]. Yet, while entropically beneficial, this conformational restriction may limit the diversity of paratopes that a library can present. Moreover, it eliminates the benefits of cysteine-free ligands: intracellular use, efficient cytoplasmic production in bacteria, and genetically introduced cysteines for site-specific thiol chemistry.

In conclusion, the extent of diversity and particular amino acid distributions consistent with a broad capacity for evolution of new binding activity were determined for a combinatorial library of a hydrophilic fibronectin domain. A gradient of diversity including sitewise constraints was revealed in evolved clones, which is consistent with natural antibody repertoires but converse to current synthetic scaffold combinatorial library designs. Importantly, the extensive dataset allowed for initial characterization of a broadly applicable data driven library design model that guides the most beneficial distribution of amino acids at each position.

## Supporting Information

S1 Fig Schematic of Study. Ligands to six different targets are generated using yeast display of Fn3HP mutants with magnetic and fluorescence selections. Deep sequencing reveals functional ligand sequences. Multiple informatics analyses indicate sitewise amino acid frequencies and their implications, relative evolutionary fitness of constrained library design, and the correlation of constrained designs with computable parameters.
(TIF)

S2 Fig Binder selection via fluorescence activated cell sorting (FACS). Diverse populations of evolved clones were isolated via cytometry. Two representative campaigns, goat IgG and lysozyme are shown (left column) with 50 nM multivalent target (3:1 stoichiometry of target preloaded on streptavidin-AlexaFluor488). Specificity controls (right column) were conducted under identical multivalent labeling conditions where non-cognate proteins lysozyme and rabbit IgG were incubated with the evolved goat IgG and lysozyme populations, respectively. Binding clones within the gated region (solid line) were isolated from each target sample for further analysis. The analogous gated regions (dashed lines) within the control samples are shown for comparison.
(TIF)

S3 Fig Correlative parametric analysis of amino acid distributions. Sitewise evaluation of theoretical stability upon mutation and natural sequence frequency, as well as overall amino acid prevalence at binding interfaces of antibodies (i.e. complementarity), generate sitewise amino acid frequencies. The ability of these frequencies–scaled linearly based on solvent exposure and target exposure (Eq 1)–to collectively mimic the observed sitewise amino acid distributions in binding populations is evaluated. The optimal weights for each contributing data set as a function of exposure are shown. (A-C) For the indicated weights of each metric, the other free parameters were varied to optimize the match between modeled sitewise amino acid distributions and experimentally observed sequences. The qualities of the fits are presented as the number of standard deviations above the fit obtained if unbiased data are used (*i*.*e*. uniformly 5% amino acid diversity rather than stability, homology, and complementarity bias). (A) Relative success when limited to two data inputs. Exposure independent (α) and dependent (β) weights are varied, subject to the indicated average weight, to maximize fit. (B) Sensitivity of exposure independent weights (α). All αvalues are fixed as indicated (note that all α’s sum to 1 so complementarity weight is implicit). Exposure dependent weights are varied to maximize fit. 55% complementarity, 45% natural sequence frequency, and 0% theoretical stability optimize fit. (C) As in (B) but with set βvalues and varied αvalues.
(TIF)

S1 Table Hydrophilic fibronectin (Fn3HP) sequence information and library oligonucleotides. (A) Fn3HP framework amino acid and DNA sequence. All framework sites are conserved as the sequence of the tenth type III domain of human fibronectin with the hydrophilic mutations V1S, V4S, V11T, A12N, T16N, L19T, V45S, and V66Q [50], underlined, as well as the stabilizing D7N [74], shown with overbar. (B) Oligonucleotide DNA sequences used for constructing generation one library. Sequences are composed of standard nucleotides (ACGT), degenerate nucleotides (RYMKSWHBVDN), and a specialty codon mix (xyz) which uses the following nucleotide frequencies: 20% A, 15% C, 25% G, and 40% T at site 1, 50% A, 25% C, 15% G, and 10% T at site 2, and 0% A, 45% C, 10% G, and 45% T at site 3. Oligos are arranged by loop (BC, DE, FG), sublibraries a-e, and amino acid length of the diversified region within the loop.
(PDF)

S2 Table Oligonucleotide DNA sequences used for constructing generation two library. Sequences are composed of standard nucleotides (ACGT), degenerate nucleotides (RYMKSWHBVDN), and a specialty codon mix (xyz) which uses the following nucleotide frequencies: 20% A, 15% C, 25% G, and 40% T at site 1, 50% A, 25% C, 15% G, and 10% T at site 2, and 0% A, 45% C, 10% G, and 45% T at site 3. Oligos are arranged by loop (BC, DE, FG), loop specific sublibraries, and amino acid length of the diversified region within the loop.
(PDF)

S3 Table Correlative parametric analysis of amino acid distributions—input matrices. Library design can be guided by information regarding each position’s mutational tolerance and naturally evolved sequence to reduce the prevalence of overly destabilizing mutations as well as identifying structurally stabilizing mutations. Additionally, the chemical diversity found at the interfaces of well characterized natural binders, such as the complementarity determining regions (CDR) of antibodies, can be applied to protein scaffolds to accommodate for strong binding interactions. Here, a model for library design was built based on a linear combination of (A) computational stability, (B) natural homolog sequence frequency, and (C) CDR diversity input matrices. These three elements were weighted based on the (D) target exposure (i.e. proximity to the binding interface) and solvent exposed surface area (i.e. orientation and packing) of each site.
(PDF)

S4 Table Illumina primer design. Conserved framework positions with regions or sites having low diversity, as is the case with conserved framework positions, require additional considerations during sample preparation to ensure a high level of accuracy during the MiSeq run. The inclusion of variable length degenerate sequence (N_4-8_) at 5’ and 3’ ends allow the conserved sites to be offset. Based on TruSeq guidelines, adapter indices are designed to have balanced G/T and C/A content, the following 6 adapter index tags were selected: AD005,6,12,14,18,19. Schematic below demonstrates the two-step PCR used for amplicon library construction. Colored regions of schematic indicate (from left to right) TruSeq universal adapter (red), target primer (cyan), gene of interest (gray), reverse target primer (green), multiplex primer 2.0 (yellow), Illumina index (blue), and Illumina PCR primer (purple). Table at bottom lists individual sequences with item names corresponding to PCR schematic.
(PDF)

## References

[1] Romero PA, Arnold FH. Exploring protein fitness landscapes by directed evolution. Nat Rev Mol Cell Biol 2009;10:866–76. 10.1038/nrm2805 19935669 PMC2997618

[2] Arnold FH. Fancy footwork in the sequence space shuffle. Nat Biotechnol 2006;24:328–30. 10.1038/nbt0306-328 16525408

[3] Karanicolas J, Corn JE, Chen I, Joachimiak LA, Dym O, Peck SH, et al A de novo protein binding pair by computational design and directed evolution. Mol Cell 2011;42:250–60. 10.1016/j.molcel.2011.03.010 21458342 PMC3102007

[4] Fleishman SJ, Whitehead T a, Ekiert DC, Dreyfus C, Corn JE, Strauch E-M, et al Computational design of proteins targeting the conserved stem region of influenza hemagglutinin. Science 2011;332:816–21. 10.1126/science.1202617 21566186 PMC3164876

[5] Khoury G a., Smadbeck J, Kieslich C a., Floudas C a. Protein folding and de novo protein design for biotechnological applications. Trends Biotechnol 2014;32:99–109. 10.1016/j.tibtech.2013.10.008 24268901 PMC3922204

[6] Dellus-Gur E, Toth-Petroczy A, Elias M, Tawfik DS. What makes a protein fold amenable to functional innovation? fold polarity and stability trade-offs. J Mol Biol 2013;425:2609–21. 10.1016/j.jmb.2013.03.033 23542341

[7] Tokuriki N, Tawfik DS. Stability effects of mutations and protein evolvability. Curr Opin Struct Biol 2009;19:596–604. 10.1016/j.sbi.2009.08.003 19765975

[8] Stern L, Case B, Hackel B. Alternative non-antibody protein scaffolds for molecular imaging of cancer. Curr Opin Chem Eng 2013. 10.1016/j.coche.2013.08.009 PMC3863941 24358455

[9] Chen J, Sawyer N, Regan L. Protein-protein interactions: General trends in the relationship between binding affinity and interfacial buried surface area. Protein Sci 2013;22:510–5. 10.1002/pro.2230 23389845 PMC3610057

[10] Zhai W, Glanville J, Fuhrmann M, Mei L, Ni I, Sundar PD, et al Synthetic antibodies designed on natural sequence landscapes. J Mol Biol 2011;412:55–71. 10.1016/j.jmb.2011.07.018 21787786

[11] Knappik A, Ge L, Honegger A, Pack P, Fischer M, Wellnhofer G, et al Fully synthetic human combinatorial antibody libraries (HuCAL) based on modular consensus frameworks and CDRs randomized with trinucleotides. J Mol Biol 2000;296:57–86. 10.1006/jmbi.1999.3444 10656818

[12] Prassler J, Thiel S, Pracht C, Polzer A, Peters S, Bauer M, et al HuCAL PLATINUM, a synthetic fab library optimized for sequence diversity and superior performance in mammalian expression systems. J Mol Biol 2011;413:261–78. 10.1016/j.jmb.2011.08.012 21856311

[13] Sidhu SS, Li B, Chen Y, Fellouse FA, Eigenbrot C, Fuh G. Phage-displayed antibody libraries of synthetic heavy chain complementarity determining regions. J Mol Biol 2004;338:299–310. 15066433 10.1016/j.jmb.2004.02.050

[14] Fellouse FA, Esaki K, Birtalan S, Raptis D, Cancasci VJ, Koide A, et al High-throughput Generation of Synthetic Antibodies from Highly Functional Minimalist Phage-displayed Libraries. J Mol Biol 2007;373:924–40. 10.1016/j.jmb.2007.08.005 17825836

[15] Grönwall C, Jonsson A, Lindström S, Gunneriusson E, Ståhl S, Herne N. Selection and characterization of Affibody ligands binding to Alzheimer amyloid beta peptides. J Biotechnol 2007;128:162–83. 17088007 10.1016/j.jbiotec.2006.09.013

[16] Correa A, Pacheco S, Mechaly AE, Obal G, Béhar G, Mouratou B, et al Potent and specific inhibition of glycosidases by small artificial binding proteins (Affitins). PLoS One 2014;9 10.1371/journal.pone.0097438 PMC4019568 24823716

[17] Béhar G, Bellinzoni M, Maillasson M, Paillard-Laurance L, Alzari PM, He X, et al Tolerance of the archaeal Sac7d scaffold protein to alternative library designs: characterization of anti-immunoglobulin G Affitins. Protein Eng Des Sel 2013;26:267–75. 10.1093/protein/gzs106 23315487

[18] Getz J a., Rice JJ, Daugherty PS. Protease-resistant peptide ligands from a knottin scaffold library. ACS Chem Biol 2011;6:837–44. 10.1021/cb200039s 21615106 PMC3158827

[19] Moore SJ, Cochran JR. Engineering knottins as novel binding agents. Methods Enzymol 2012;503:223–51. 10.1016/B978-0-12-396962-0.00009-4 22230571

[20] Gebauer M, Schiefner A, Matschiner G, Skerra A. Combinatorial Design of an Anticalin Directed against the Extra-Domain B for the Specific Targeting of Oncofetal Fibronectin. J Mol Biol 2013;425:780–802. 10.1016/j.jmb.2012.12.004 23238252

[21] Schlatter D, Brack S, Banner DW, Batey S, Benz J, Bertschinger J, et al Generation, characterization and structural data of chymase binding proteins based on the human Fyn kinase SH3 domain. MAbs 2012;4:497–508. 10.4161/mabs.20452 22653218 PMC3499344

[22] Gera N, Hussain M, Wright RC, Rao BM. Highly stable binding proteins derived from the hyperthermophilic Sso7d scaffold. J Mol Biol 2011;409:601–16. 10.1016/j.jmb.2011.04.020 21515282

[23] Steemson JD, Baake M, Rakonjac J, Arcus VL, Liddament MT. Tracking Molecular Recognition at the Atomic Level with a New Protein Scaffold Based on the OB-Fold. PLoS One 2014;9:e86050 10.1371/journal.pone.0086050 24465865 PMC3896448

[24] Barbas CF, Bain JD, Hoekstra DM, Lerner RA. Semisynthetic combinatorial antibody libraries: a chemical solution to the diversity problem. Proc Natl Acad Sci U S A 1992;89:4457–61. 10.1073/pnas.89.10.4457 1584777 PMC49101

[25] Birtalan S, Fisher RD, Sidhu SS. The functional capacity of the natural amino acids for molecular recognition. Mol Biosyst 2010;6:1186–94. 10.1039/b927393j 20383388

[26] Fellouse FA, Wiesmann C, Sidhu SS. Synthetic antibodies from a four-amino-acid code: a dominant role for tyrosine in antigen recognition. Proc Natl Acad Sci U S A 2004;101:12467–72. 10.1073/pnas.0401786101 15306681 PMC515084

[27] Binz HK, Amstutz P, Kohl A, Stumpp MT, Briand C, Forrer P, et al High-affinity binders selected from designed ankyrin repeat protein libraries. Nat Biotechnol 2004;22:575–82. 15097997 10.1038/nbt962

[28] Seeger MA, Zbinden R, Flütsch A, Gutte PGM, Engeler S, Roschitzki-Voser H, et al Design, construction, and characterization of a second-generation DARPin library with reduced hydrophobicity. Protein Sci 2013;22:1239–57. 10.1002/pro.2312 23868333 PMC3776336

[29] Koide A, Bailey CW, Huang X, Koide S. The fibronectin type III domain as a scaffold for novel binding proteins. J Mol Biol 1998;284:1141–51. 10.1006/jmbi.1998.2238 9837732

[30] Lipovsek D. Adnectins: engineered target-binding protein therapeutics. Protein Eng Des Sel 2011;24:3–9. 10.1093/protein/gzq097 21068165 PMC3003446

[31] Koide A, Wojcik J, Gilbreth RN, Hoey RJ, Koide S. Teaching an old scaffold new tricks: Monobodies constructed using alternative surfaces of the FN3 scaffold. J Mol Biol 2012;415:393–405. 10.1016/j.jmb.2011.12.019 22198408 PMC3260337

[32] Diem MD, Hyun L, Yi F, Hippensteel R, Kuhar E, Lowenstein C, et al Selection of high-affinity Centyrin FN3 domains from a simple library diversified at a combination of strand and loop positions. Protein Eng Des Sel 2014 10.1093/protein/gzu016 24786107

[33] Wojcik J, Hantschel O, Grebien F, Kaupe I, Bennett KL, Barkinge J, et al A potent and highly specific FN3 monobody inhibitor of the Abl SH2 domain. Nat Struct Mol Biol 2010;17:519–27. 10.1038/nsmb.1793 20357770 PMC2926940

[34] Koide A, Wojcik J, Gilbreth RN, Reichel A, Piehler J, Koide S. Accelerating phage-display library selection by reversible and site-specific biotinylation. Protein Eng Des Sel 2009;22:685–90. 10.1093/protein/gzp053 19737805 PMC2763796

[35] Hackel BJ, Ackerman ME, Howland SW, Wittrup KD. Stability and CDR Composition Biases Enrich Binder Functionality Landscapes. J Mol Biol 2010;401:84–96. 10.1016/j.jmb.2010.06.004 20540948 PMC3927142

[36] Lipovšek D, Lippow SM, Hackel BJ, Gregson MW, Cheng P, Kapila A, et al Evolution of an Interloop Disulfide Bond in High-Affinity Antibody Mimics Based on Fibronectin Type III Domain and Selected by Yeast Surface Display: Molecular Convergence with Single-Domain Camelid and Shark Antibodies. J Mol Biol 2007;368:1024–41. 10.1016/j.jmb.2007.02.029 17382960

[37] Hackel BJ, Kapila A, Dane Wittrup K. Picomolar Affinity Fibronectin Domains Engineered Utilizing Loop Length Diversity, Recursive Mutagenesis, and Loop Shuffling. J Mol Biol 2008;381:1238–52. 10.1016/j.jmb.2008.06.051 18602401 PMC2840393

[38] Sullivan M, Brooks L, Weidenborner P. Anti-Idiotypic Monobodies Derived from a Fibronectin Scaffold. Biochemistry 2013;52:1802–13. 10.1021/bi3016668 23394681 PMC4090420

[39] Liao H-I, Olson CA, Hwang S, Deng H, Wong E, Baric RS, et al mRNA display design of fibronectin-based intrabodies that detect and inhibit sars-cov N protein. J Biol Chem 2009;284:M901547200 10.1074/jbc.M901547200 PMC2719390 19364769

[40] Gilbreth RN, Truong K, Madu I, Koide A, Wojcik JB, Li N-S, et al Isoform-specific monobody inhibitors of small ubiquitin-related modifiers engineered using structure-guided library design. Proc Natl Acad Sci U S A 2011;108:7751–6. 10.1073/pnas.1102294108 21518904 PMC3093456

[41] Tamaskovic R, Simon M, Stefan N, Schwill M, Plückthun A. Designed ankyrin repeat proteins (DARPins): From research to therapy. Methods Enzymol 2012;503:101–34. 10.1016/B978-0-12-396962-0.00005-7 22230567

[42] Grimm S, Yu F, Nygren PÅ. Ribosome display selection of a murine IgG1 fab binding affibody molecule allowing species selective recovery of monoclonal antibodies. Mol Biotechnol 2011;48:263–76. 10.1007/s12033-010-9367-1 21197589 PMC3115053

[43] Gebauer M, Skerra A. Anticalins: Small engineered binding proteins based on the lipocalin scaffold. Methods Enzymol 2012;503:157–88. 10.1016/B978-0-12-396962-0.00007-0 22230569

[44] Reichmann D, Cohen M, Abramovich R, Dym O, Lim D, Strynadka NCJ, et al Binding Hot Spots in the TEM1-BLIP Interface in Light of its Modular Architecture. J Mol Biol 2007;365:663–79. 10.1016/j.jmb.2006.09.076 17070843

[45] Schreiber G, Fersht AR. Energetics of protein-protein interactions: Analysis ofthe Barnase-Barstar interface by single mutations and double mutant cycles. J Mol Biol 1995;248:478–86. 10.1016/S0022-2836(95)80064-6 7739054

[46] Dall’Acqua W, Goldman ER, Eisenstein E, Mariuzza RA. A Mutational Analysis of the Binding of Two Different Proteins to the Same Antibody. Biochemistry 1996;35:9667–76. 10.1021/bi960819i 8703938

[47] Cunningham BC, Wells JA. Comparison of a structural and a functional epitope. J Mol Biol 1993;234:554–63. 10.1006/jmbi.1993.1611 7504735

[48] Clackson T, Wells JA. A hot spot of binding energy in a hormone-receptor interface. Science (80-) 1995;267:383–6. 10.1126/science.7529940 7529940

[49] Jones JT. Binding Interaction of the Heregulinbeta egf Domain with ErbB3 and ErbB4 Receptors Assessed by Alanine Scanning Mutagenesis. J Biol Chem 1998;273:11667–74. 10.1074/jbc.273.19.11667 9565587

[50] Hackel BJ, Sathirachinda A, Gambhir SS. Designed hydrophilic and charge mutations of the fibronectin domain: Towards tailored protein biodistribution. Protein Eng Des Sel 2012;25:639–47. 10.1093/protein/gzs036 22691700 PMC3449399

[51] Bogan AA, Thorn KS. Anatomy of hot spots in protein interfaces. J Mol Biol 1998;280:1–9. 10.1006/jmbi.1998.1843 9653027

[52] DeLano WL. Unraveling hot spots in binding interfaces: progress and challenges. Curr Opin Struct Biol 2002;12:14–20. 10.1016/S0959-440X(02)00283-X 11839484

[53] Whitehead TA, Chevalier A, Song Y, Dreyfus C, Fleishman SJ, De Mattos C, et al Optimization of affinity, specificity and function of designed influenza inhibitors using deep sequencing. Nat Biotechnol 2012;30:543–8. 10.1038/nbt.2214 22634563 PMC3638900

[54] Ernst A, Gfeller D, Kan Z, Seshagiri S, Kim PM, Bader GD, et al Coevolution of PDZ domain-ligand interactions analyzed by high-throughput phage display and deep sequencing. Mol Biosyst 2010;6:1782–90. 10.1039/c0mb00061b 20714644

[55] Deng Z, Huang W, Bakkalbasi E, Brown NG, Adamski CJ, Rice K, et al Deep sequencing of systematic combinatorial libraries reveals?? -lactamase sequence constraints at high resolution. J Mol Biol 2012;424:150–67. 10.1016/j.jmb.2012.09.014 23017428 PMC3524589

[56] Ravn U, Gueneau F, Baerlocher L, Osteras M, Desmurs M, Malinge P, et al By-passing in vitro screening—Next generation sequencing technologies applied to antibody display and in silico candidate selection. Nucleic Acids Res 2010;38 10.1093/nar/gkq789 PMC2995085 20846958

[57] Boder ET, Wittrup KD. Yeast surface display for screening combinatorial polypeptide libraries. Nat Biotechnol 1997;15:553–7. 10.1038/nbt0697-553 9181578

[58] Benatuil L, Perez JM, Belk J, Hsieh C-M. An improved yeast transformation method for the generation of very large human antibody libraries (supplementary info). Protein Eng Des Sel 2010;23:9–10. 20130105 10.1093/protein/gzq002

[59] Ackerman M, Levary D, Tobon G, Hackel B, Orcutt KD, Wittrup KD. Highly avid magnetic bead capture: An efficient selection method for de novo protein engineering utilizing yeast surface display. Biotechnol Prog 2009;25:774–83. 10.1002/btpr.174 19363813 PMC2837102

[60] Chao G, Lau WL, Hackel BJ, Sazinsky SL, Lippow SM, Wittrup KD. Isolating and engineering human antibodies using yeast surface display. Nat Protoc 2006;1:755–68. 10.1038/nprot.2006.94 17406305

[61] Van Dijk EL, Jaszczyszyn Y, Thermes C. Library preparation methods for next-generation sequencing: tone down the bias. Exp Cell Res 2014;322:12–20. 10.1016/j.yexcr.2014.01.008 24440557

[62] Dabney J, Meyer M. Length and GC-biases during sequencing library amplification: a comparison of various polymerase-buffer systems with ancient and modern DNA sequencing libraries. Biotechniques 2012;52:87–94. 10.2144/000113809 22313406

[63] Masella AP, Bartram AK, Truszkowski JM, Brown DG, Neufeld JD. PANDAseq: paired-end assembler for illumina sequences. BMC Bioinformatics 2012;13:31 10.1186/1471-2105-13-31 22333067 PMC3471323

[64] Sanner M. Python: a programming language for software integration and development. J Mol Graph Model 1999;17:57–61. 10660911

[65] Greenfield NJ. Using circular dichroism spectra to estimate protein secondary structure. Nat Protoc 2006;1:2876–90. 10.1038/nprot.2006.202 17406547 PMC2728378

[66] Xu L, Aha P, Gu K, Kuimelis RG, Kurz M, Lam T, et al Directed evolution of high-affinity antibody mimics using mRNA display. Chem Biol 2002;9:933–42. 10.1016/S1074-5521(02)00187-4 12204693

[67] Sonnhammer ELL, Eddy SR, Durbin R. Pfam: A comprehensive database of protein domain families based on seed alignments. Proteins Struct Funct Genet 1997;28:405–20. 10.1002/(SICI)1097-0134(199707)28:3<405::AID-PROT10>3.0.CO;2-L 9223186

[68] Schymkowitz J, Borg J, Stricher F, Nys R, Rousseau F, Serrano L. The FoldX web server: an online force field. Nucleic Acids Res 2005;33:W382–8. 10.1093/nar/gki387 15980494 PMC1160148

[69] Berman HM, Westbrook J, Feng Z, Gilliland G, Bhat TN, Weissig H, et al The Protein Data Bank 2000;28:235–42. 10592235 10.1093/nar/28.1.235 PMC102472

[70] Cota E, Hamill SJ, Fowler SB, Clarke J. Two proteins with the same structure respond very differently to mutation: the role of plasticity in protein stability. J Mol Biol 2000;302:713–25. 10.1006/jmbi.2000.4053 10986129

[71] Fellouse FA, Barthelemy PA, Kelley RF, Sidhu SS. Tyrosine Plays a Dominant Functional Role in the Paratope of a Synthetic Antibody Derived from a Four Amino Acid Code. J Mol Biol 2006;357:100–14. 10.1016/j.jmb.2005.11.092 16413576

[72] Birtalan S, Zhang Y, Fellouse FA, Shao L, Schaefer G, Sidhu SS. The Intrinsic Contributions of Tyrosine, Serine, Glycine and Arginine to the Affinity and Specificity of Antibodies. J Mol Biol 2008;377:1518–28. 10.1016/j.jmb.2008.01.093 18336836

[73] Koide S, Sidhu SS. The importance of being tyrosine: lessons in molecular recognition from minimalist synthetic binding proteins. ACS Chem Biol 2009;4:325–34. 10.1021/cb800314v 19298050 PMC2829252

[74] Koide A, Jordan MR, Horner SR, Batori V, Koide S. Stabilization of a Fibronectin Type III Domain by the Removal of Unfavorable Electrostatic Interactions on the Protein Surface. Biochemistry 2001;40:10326–33. 10.1021/bi010916y 11513611

[75] Shannon CE. A mathematical theory of communication. Bell Syst Tech J 1948;27:623–56.

[76] Henikoff S, Henikoff JG. Amino acid substitution matrices from protein blocks. Proc Natl Acad Sci U S A 1992;89:10915–9. 10.1073/pnas.89.22.10915 1438297 PMC50453

[77] Steipe B, Schiller B, Pluckthun A, Steinbacher S. Sequence statistics reliably predict stabilizing mutations in a protein domain. J Mol Biol 1994;240:188–92. 8028003 10.1006/jmbi.1994.1434

[78] Clark JM. Novel non-templated nucleotide addition reactions catalyzed by procaryotic and eucaryotic DNA polymerases. Nucleic Acids Res 1988;16:9677–86. 10.1093/nar/16.20.9677 2460825 PMC338772

[79] Wootton JC, Federhen S. Statistics of local complexity in amino acid sequences and sequence databases. Comput Chem 1993;17:149–63. 10.1016/0097-8485(93)85006-X

[80] Parker MH, Chen Y, Danehy F, Dufu K, Ekstrom J, Getmanova E, et al Antibody mimics based on human fibronectin type three domain engineered for thermostability and high-affinity binding to vascular endothelial growth factor receptor two. Protein Eng Des Sel 2005;18:435–44. 10.1093/protein/gzi050 16087651

[81] Traxlmayr MW, Hasenhindl C, Hackl M, Stadlmayr G, Rybka JD, Borth N, et al Construction of a stability landscape of the CH3 domain of human IgG1 by combining directed evolution with high throughput sequencing. J Mol Biol 2012;423:397–412. 10.1016/j.jmb.2012.07.017 22846908 PMC3469823

[82] Finn RD, Bateman A, Clements J, Coggill P, Eberhardt RY, Eddy SR, et al Pfam: The protein families database. Nucleic Acids Res 2014;42:D290–301. 10.1093/nar/gkt1223

[83] Zemlin M, Klinger M, Link J, Zemlin C, Bauer K, Engler JA, et al Expressed Murine and Human CDR-H3 Intervals of Equal Length Exhibit Distinct Repertoires that Differ in their Amino Acid Composition and Predicted Range of Structures. J Mol Biol 2003;334:733–49. 10.1016/j.jmb.2003.10.007 14636599

[84] Fraczkiewicz R, Braun W. Exact and efficient analytical calculation of the accessible surface areas and their gradients for macromolecules. J Comput Chem 1998;19:319–33. 10.1002/(SICI)1096-987X(199802)19:3<319::AID-JCC6>3.0.CO;2-W

[85] Pal G, Kossiakoff AA, Sidhu SS. The functional binding epitope of a high affinity variant of human growth hormone mapped by shotgun alanine-scanning mutagenesis: insights into the mechanisms responsible for improved affinity. J Mol Biol 2003;332:195–204. 12946357 10.1016/s0022-2836(03)00898-2

[86] Ma B, Wolfson HJ, Nussinov R. Protein functional epitopes: Hot spots, dynamics and combinatorial libraries. Curr Opin Struct Biol 2001;11:364–9. 10.1016/S0959-440X(00)00216-5 11406388

[87] Kortemme T, Baker D. A simple physical model for binding energy hot spots in protein-protein complexes. Proc Natl Acad Sci U S A 2002;99:14116–21. 10.1073/pnas.202485799 12381794 PMC137846

[88] Daugherty PS, Chen G, Iverson BL, Georgiou G. Quantitative analysis of the effect of the mutation frequency on the affinity maturation of single chain Fv antibodies. Proc Natl Acad Sci U S A 2000;97:2029–34. 10.1073/pnas.030527597 10688877 PMC15748

[89] Guo HH, Choe J, Loeb LA. Protein tolerance to random amino acid change. Proc Natl Acad Sci U S A 2004;101:9205–10. 10.1073/pnas.0403255101 15197260 PMC438954

[90] Shafikhani S, Siegel RA, Ferrari E, Schellenberger V. Generation of large libraries of random mutants in Bacillus subtills by PCR-based plasmid multimerization. Biotechniques 1997;23:304–10. 9266088 10.2144/97232rr01

[91] Fellouse FA, Li B, Compaan DM, Peden AA, Hymowitz SG, Sidhu SS. Molecular recognition by a binary code. J Mol Biol 2005;348:1153–62. 10.1016/j.jmb.2005.03.041 15854651

[92] Walensky LD, Kung AL, Escher I, Malia TJ, Barbuto S, Wright RD, et al Activation of apoptosis in vivo by a hydrocarbon-stapled BH3 helix. Science 2004;305:1466–70. 10.1126/science.1099191 15353804 PMC1360987

[93] Roux KH, Greenberg AS, Greene L, Strelets L, Avila D, McKinney EC, et al Structural analysis of the nurse shark (new) antigen receptor (NAR): molecular convergence of NAR and unusual mammalian immunoglobulins. Proc Natl Acad Sci U S A 1998;95:11804–9. 10.1073/pnas.95.20.11804 9751746 PMC21721

[94] Muyldermans S, Atarhouch T, Saldanha J, Barbosa JA, Hamers R. Sequence and structure of VH domain from naturally occurring camel heavy chain immunoglobulins lacking light chains. Protein Eng 1994;7:1129–35. 10.1093/protein/7.9.1129 7831284

