# Supplementary figures and images for: High-Throughput Ligand Discovery Reveals a Sitewise Gradient of Diversity in Broadly Evolved Hydrophilic Fibronectin Domains
Source: PLoS One. 2015 Sep 18;10(9):e0138956. doi: 10.1371/journal.pone.0138956 (PMC4575168; doi:10.1371/journal.pone.0138956)

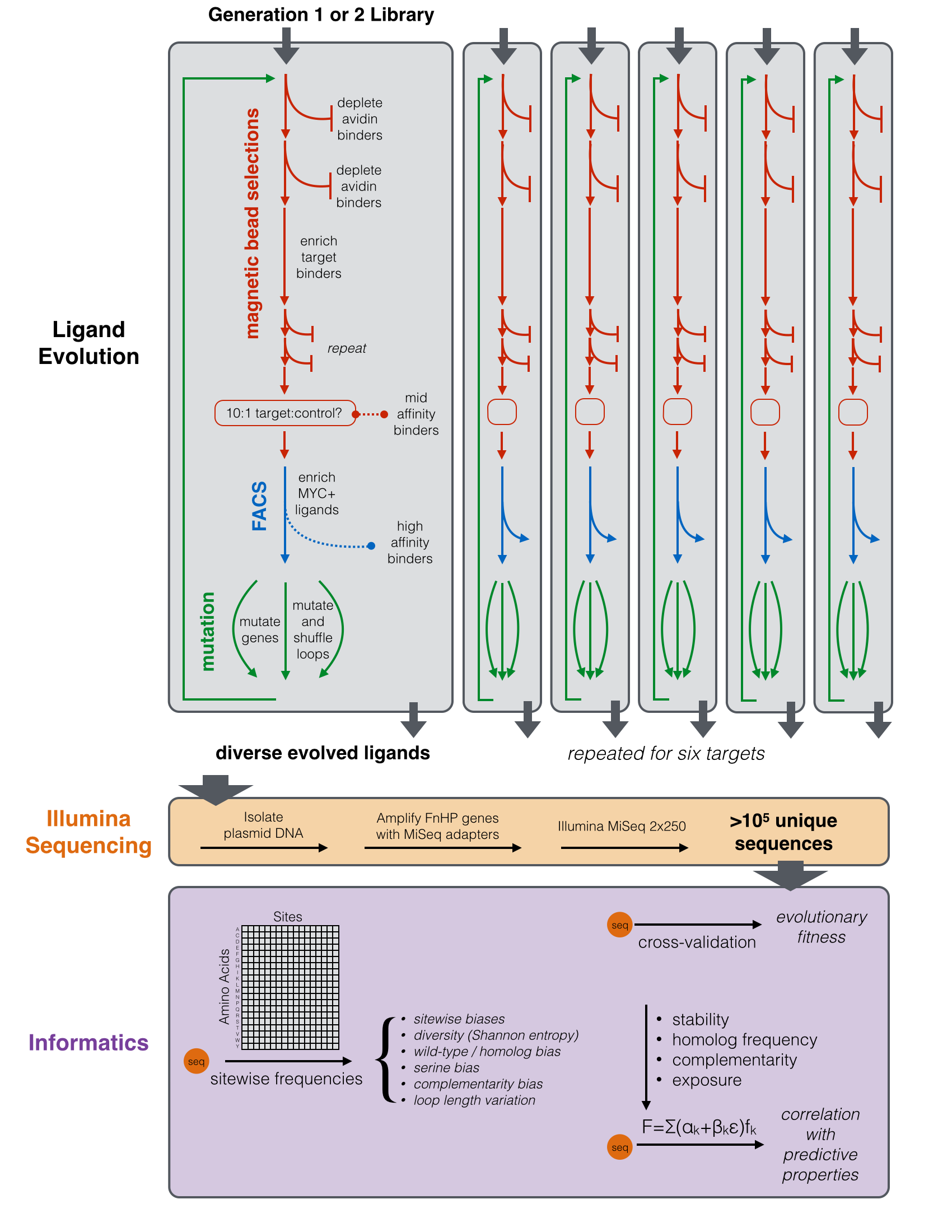

Supplement: S1 Fig — Ligands to six different targets are generated using yeast display of Fn3HP mutants with magnetic and fluorescence selections. Deep sequencing reveals functional ligand sequences. Multiple informatics analyses indicate sitewise amino acid frequencies and their implications, relative evolutionary fitness of constrained library design, and the correlation of constrained designs with computable parameters. (TIF) [file pone.0138956.s001.tif]

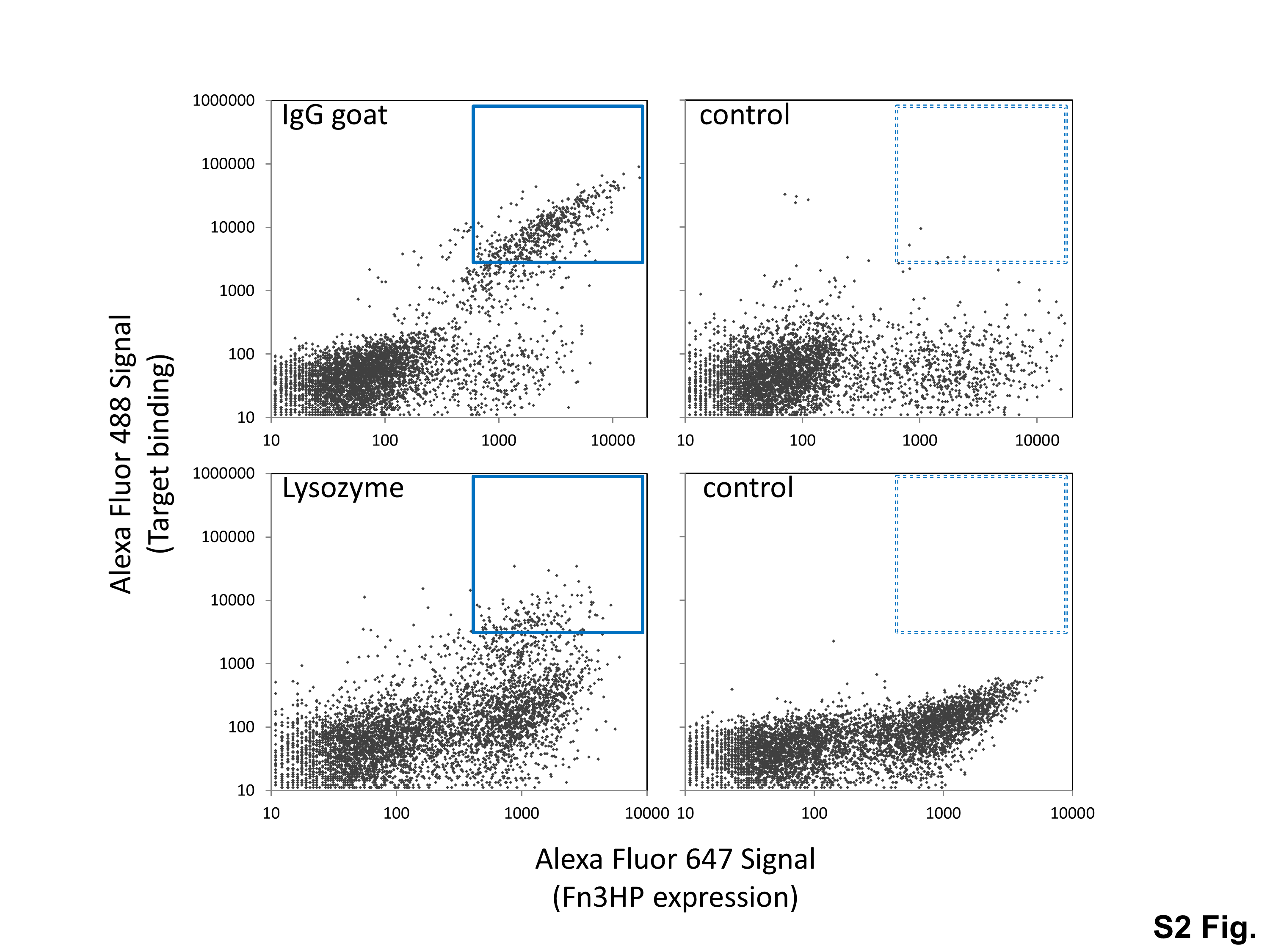

Supplement: S2 Fig — Diverse populations of evolved clones were isolated via cytometry. Two representative campaigns, goat IgG and lysozyme are shown (left column) with 50 nM multivalent target (3:1 stoichiometry of target preloaded on streptavidin-AlexaFluor488). Specificity controls (right column) were conducted under identical multivalent labeling conditions where non-cognate proteins lysozyme and rabbit IgG were incubated with the evolved goat IgG and lysozyme populations, respectively. Binding clones within the gated region (solid line) were isolated from each target sample for further analysis. The analogous gated regions (dashed lines) within the control samples are shown for comparison. (TIF) [file pone.0138956.s002.tif]

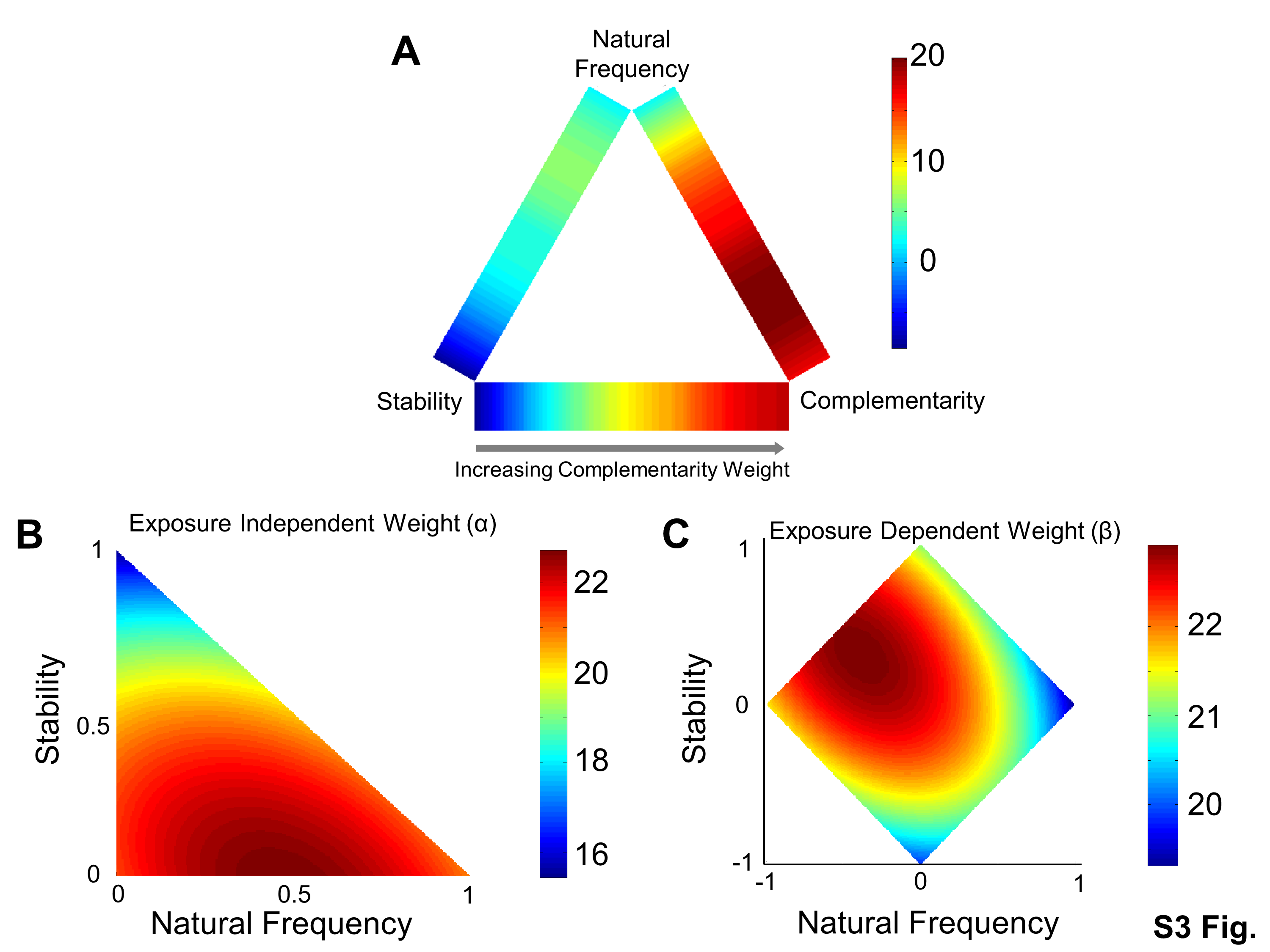

Supplement: S3 Fig — Sitewise evaluation of theoretical stability upon mutation and natural sequence frequency, as well as overall amino acid prevalence at binding interfaces of antibodies (i.e. complementarity), generate sitewise amino acid frequencies. The ability of these frequencies–scaled linearly based on solvent exposure and target exposure (Eq 1)–to collectively mimic the observed sitewise amino acid distributions in binding populations is evaluated. The optimal weights for each contributing data set as a function of exposure are shown. (A-C) For the indicated weights of each metric, the other free parameters were varied to optimize the match between modeled sitewise amino acid distributions and experimentally observed sequences. The qualities of the fits are presented as the number of standard deviations above the fit obtained if unbiased data are used (i.e. uniformly 5% amino acid diversity rather than stability, homology, and complementarity bias). (A) Relative success when limited to two data inputs. Exposure independent (α) and dependent (β) weights are varied, subject to the indicated average weight, to maximize fit. (B) Sensitivity of exposure independent weights (α). All αvalues are fixed as indicated (note that all α’s sum to 1 so complementarity weight is implicit). Exposure dependent weights are varied to maximize fit. 55% complementarity, 45% natural sequence frequency, and 0% theoretical stability optimize fit. (C) As in (B) but with set βvalues and varied αvalues. (TIF) [file pone.0138956.s003.tif]
