# Supplementary material for: High-Throughput Ligand Discovery Reveals a Sitewise Gradient of Diversity in Broadly Evolved Hydrophilic Fibronectin Domains
Source: PLoS One. 2015 Sep 18;10(9):e0138956. doi: 10.1371/journal.pone.0138956 (PMC4575168; doi:10.1371/journal.pone.0138956)
Supplement: S1 Table — (A) Fn3HP framework amino acid and DNA sequence. All framework sites are conserved as the sequence of the tenth type III domain of human fibronectin with the hydrophilic mutations V1S, V4S, V11T, A12N, T16N, L19T, V45S, and V66Q [50], underlined, as well as the stabilizing D7N [74], shown with overbar. (B) Oligonucleotide DNA sequences used for constructing generation one library. Sequences are composed of standard nucleotides (ACGT), degenerate nucleotides (RYMKSWHBVDN), and a specialty codon mix (xyz) which uses the following nucleotide frequencies: 20% A, 15% C, 25% G, and 40% T at site 1, 50% A, 25% C, 15% G, and 10% T at site 2, and 0% A, 45% C, 10% G, and 45% T at site 3. Oligos are arranged by loop (BC, DE, FG), sublibraries a-e, and amino acid length of the diversified region within the loop. (PDF) [file pone.0138956.s004.pdf]

S1 Table. Hydrophilic fibronectin (Fn3HP) sequence information and library oligonucleotides.

(A) Fn3HP framework amino acid and DNA sequence. All framework sites are conserved as the sequence of the tenth type III domain of human fibronectin with the hydrophilic mutations V1S, V4S, V11T, A12N, T16N, L19T, V45S, and V66Q [39], underlined, as well as the stabilizing D7N [63], shown with overbar.

```
SSDSPRNLEV TNATPNSLTI SWxxxxxxxx xYRITYGETG GNSPSQEFTV PxxxxxATIS GLKPGQDYTI TVYAVxxxxx
xxxxxxPISI NYRTEIDKPS Q
```

```
TCC TCC GAC TCT CCG CGT AAC CTG GAG GTT ACC AAC GCA ACT CCG AAC TCT CTG ACT ATT TCT TGG NNN NNN
NNN NNN NNN NNN NNN NNN NNN TAC CGT ATC ACC TAC GGC GAA ACT GGT GGT AAC TCC CCG AGC CAG GAA TTC
ACT GTT CCG NNN NNN NNN NNN NNN GCG ACC ATC AGC GGT CTG AAA CCG GGC CAG GAT TAT ACC ATT ACC GTG
TAC GCT GTA NNN NNN NNN NNN NNN NNN NNN NNN NNN NNN NNN CCA ATC AGC ATC AAT TAT CGC ACC GAA ATC
GAC AAA CCG TCT CAG
```

(B) Oligonucleotide DNA sequences used for constructing generation one library. Sequences are composed of standard nucleotides (ACGT), degenerate nucleotides (RYMKSWHBVDN), and a specialty codon mix (xyz) which uses the following nucleotide frequencies : 20% A, 15% C, 25% G, and 40% T at site 1, 50% A, 25% C, 15% G, and 10% T at site 2, and 0% A, 45% C, 10% G, and 45% T at site 3. Oligos are arranged by loop (BC, DE, FG), sublibraries a-e, and amino acid length of the diversified region within the loop.

Loop/Sublib/Len (AA)	Oligo Sequence
BC/a/10	ACTCTCTGACTATTTCTTGGGACGCACCAxyzxyzxyzxyzDCTxyzGGATACCGTATCACCTACGGCGAAAC
BC/b/10	ACTCTCTGACTATTTCTTGGKMTKCCYCCxyzxyzxyzxyzDCTxyzRGCTACCGTATCACCTACGGCGAAAC
BC/c/10	ACTCTCTGACTATTTCTTGGKMCKMTYMTxyzxyzxyzxyzDCTxyzDRTTACCGTATCACCTACGGCGAAAC
BC/d/10	ACTCTCTGACTATTTCTTGGDVTDVTDVTxyzxyzxyzxyzDCTxyzDVTTACCGTATCACCTACGGCGAAAC
BC/e/10	ACTCTCTGACTATTTCTTGGxyzxyzxyzxyzxyzxyzxyzDCTxyzxyzTACCGTATCACCTACGGCGAAAC
BC/a/9	ACTCTCTGACTATTTCTTGGGACGCACCAxyzxyzxyzDCTxyzGGATACCGTATCACCTACGGCGAAAC
BC/b/9	ACTCTCTGACTATTTCTTGGKMTKCCYCCxyzxyzxyzDCTxyzRGCTACCGTATCACCTACGGCGAAAC
BC/c/9	ACTCTCTGACTATTTCTTGGKMCKMTYMTxyzxyzxyzDCTxyzDRTTACCGTATCACCTACGGCGAAAC
BC/d/9	ACTCTCTGACTATTTCTTGGDVTDVTDVTxyzxyzxyzDCTxyzDVTTACCGTATCACCTACGGCGAAAC
BC/e/9	ACTCTCTGACTATTTCTTGGxyzxyzxyzxyzxyzxyzDCTxyzxyzTACCGTATCACCTACGGCGAAAC
BC/a/8	ACTCTCTGACTATTTCTTGGGACGCACCAxyzxyzDCTxyzGGATACCGTATCACCTACGGCGAAAC
BC/b/8	ACTCTCTGACTATTTCTTGGKMTKCCYCCxyzxyzDCTxyzRGCTACCGTATCACCTACGGCGAAAC

BC/c/8	ACTCTCTGACTATTTCTTGGKMCKMTYMTxyzxyzDCTxyzDRTTACCGTATCACCTACGGCGAAAC
BC/d/8	ACTCTCTGACTATTTCTTGGDVTDVTDVTxyzxyzDCTxyzDVTTACCGTATCACCTACGGCGAAAC
BC/e/8	ACTCTCTGACTATTTCTTGGxyzxyzxyzxyzxyzDCTxyzxyzTACCGTATCACCTACGGCGAAAC
BC/a/7	ACTCTCTGACTATTTCTTGGGACGCACCAxyzDCTxyzGGATACCGTATCACCTACGGCGAAAC
BC/b/7	ACTCTCTGACTATTTCTTGGKMTKCCYCCxyzDCTxyzRGCTACCGTATCACCTACGGCGAAAC
BC/c/7	ACTCTCTGACTATTTCTTGGKMCKMTYMTxyzDCTxyzDRTTACCGTATCACCTACGGCGAAAC
BC/d/7	ACTCTCTGACTATTTCTTGGDVTDVTDVTxyzDCTxyzDVTTACCGTATCACCTACGGCGAAAC
BC/e/7	ACTCTCTGACTATTTCTTGGxyzxyzxyzxyzDCTxyzxyzTACCGTATCACCTACGGCGAAAC
DE/a/6	CGAGCCAGGAATTCACTGTTCCGGGATCATCAAACTCAACAGCGACCATCAGCGGTCTGAAAC
DE/b/6	CGAGCCAGGAATTCACTGTTCCGRGCTCCTCCARTTCCASCGCGACCATCAGCGGTCTGAAAC
DE/c/6	CGAGCCAGGAATTCACTGTTCCGDRTWMTWMTWMTWMTWMTGCGACCATCAGCGGTCTGAAAC
DE/d/6	CGAGCCAGGAATTCACTGTTCCGDVTDVTDVTDVTDVTDVTGCGACCATCAGCGGTCTGAAAC
DE/e/6	CGAGCCAGGAATTCACTGTTCCGxyzxyzxyzxyzxyzxyzGCGACCATCAGCGGTCTGAAAC
DE/a/5	CGAGCCAGGAATTCACTGTTCCGGGATCAAACTCAACAGCGACCATCAGCGGTCTGAAAC
DE/b/5	CGAGCCAGGAATTCACTGTTCCGRGCTCCARTTCCASCGCGACCATCAGCGGTCTGAAAC
DE/c/5	CGAGCCAGGAATTCACTGTTCCGDRTWMTWMTWMTWMTGCGACCATCAGCGGTCTGAAAC
DE/d/5	CGAGCCAGGAATTCACTGTTCCGDVTDVTDVTDVTDVTGCGACCATCAGCGGTCTGAAAC
DE/e/5	CGAGCCAGGAATTCACTGTTCCGxyzxyzxyzxyzxyzGCGACCATCAGCGGTCTGAAAC
DE/a/4	CGAGCCAGGAATTCACTGTTCCGGGATCATCAACAGCGACCATCAGCGGTCTGAAAC
DE/b/4	CGAGCCAGGAATTCACTGTTCCGRGCTCCTCCASCGCGACCATCAGCGGTCTGAAAC
DE/c/4	CGAGCCAGGAATTCACTGTTCCGDRTWMTWMTWMTGCGACCATCAGCGGTCTGAAAC
DE/d/4	CGAGCCAGGAATTCACTGTTCCGDVTDVTDVTDVTGCGACCATCAGCGGTCTGAAAC
DE/e/4	CGAGCCAGGAATTCACTGTTCCGxyzxyzxyzxyzGCGACCATCAGCGGTCTGAAAC
FG/a/11	CATTACCGTGTACGCTGTAACAxyzxyzDRTxyzxyzxyzxyzxyzTCAAACCCAATCAGCATCAATTATCGCAC

FG/b/11	CATTACCGTGTACGCTGTAASCxyzxyzDRTxyzxyzxyzxyzxyzTCCARTCCAATCAGCATCAATTATCGCAC
FG/c/11	CATTACCGTGTACGCTGTAWMTxyzxyzDRTxyzxyzxyzxyzxyzWMTWMTCCAATCAGCATCAATTATCGCAC
FG/d/11	CATTACCGTGTACGCTGTADVTxyzxyzDRTxyzxyzxyzxyzxyzDVTDVTCCAATCAGCATCAATTATCGCAC
FG/e/11	CATTACCGTGTACGCTGTAxyzxyzxyzDRTxyzxyzxyzxyzxyzxyzCCAATCAGCATCAATTATCGCAC
FG/a/10	CATTACCGTGTACGCTGTAACAxyzxyzDRTxyzxyzxyzxyzTCAAACCCAATCAGCATCAATTATCGCAC
FG/b/10	CATTACCGTGTACGCTGTAASCxyzxyzDRTxyzxyzxyzxyzTCCARTCCAATCAGCATCAATTATCGCAC
FG/c/10	CATTACCGTGTACGCTGTAWMTxyzxyzDRTxyzxyzxyzxyzWMTWMTCCAATCAGCATCAATTATCGCAC
FG/d/10	CATTACCGTGTACGCTGTADVTxyzxyzDRTxyzxyzxyzxyzDVTDVTCCAATCAGCATCAATTATCGCAC
FG/e/10	CATTACCGTGTACGCTGTAxyzxyzxyzDRTxyzxyzxyzxyzxyzCCAATCAGCATCAATTATCGCAC
FG/a/9	CATTACCGTGTACGCTGTAACAxyzxyzDRTxyzxyzxyzTCAAACCCAATCAGCATCAATTATCGCAC
FG/b/9	CATTACCGTGTACGCTGTAASCxyzxyzDRTxyzxyzxyzTCCARTCCAATCAGCATCAATTATCGCAC
FG/c/9	CATTACCGTGTACGCTGTAWMTxyzxyzDRTxyzxyzxyzWMTWMTCCAATCAGCATCAATTATCGCAC
FG/d/9	CATTACCGTGTACGCTGTADVTxyzxyzDRTxyzxyzxyzDVTDVTCCAATCAGCATCAATTATCGCAC
FG/e/9	CATTACCGTGTACGCTGTAxyzxyzxyzDRTxyzxyzxyzxyzCCAATCAGCATCAATTATCGCAC
FG/a/8	CATTACCGTGTACGCTGTAACAxyzxyzDRTxyzxyzTCAAACCCAATCAGCATCAATTATCGCAC
FG/b/8	CATTACCGTGTACGCTGTAASCxyzxyzDRTxyzxyzTCCARTCCAATCAGCATCAATTATCGCAC
FG/c/8	CATTACCGTGTACGCTGTAWMTxyzxyzDRTxyzxyzWMTWMTCCAATCAGCATCAATTATCGCAC
FG/d/8	CATTACCGTGTACGCTGTADVTxyzxyzDRTxyzxyzDVTDVTCCAATCAGCATCAATTATCGCAC
FG/e/8	CATTACCGTGTACGCTGTAxyzxyzxyzDRTxyzxyzxyzCCAATCAGCATCAATTATCGCAC
FG/a/7	CATTACCGTGTACGCTGTAACAxyzxyzDRTxyzTCAAACCCAATCAGCATCAATTATCGCAC
FG/b/7	CATTACCGTGTACGCTGTAASCxyzxyzDRTxyzTCCARTCCAATCAGCATCAATTATCGCAC
FG/c/7	CATTACCGTGTACGCTGTAWMTxyzxyzDRTxyzWMTWMTCCAATCAGCATCAATTATCGCAC
FG/d/7	CATTACCGTGTACGCTGTADVTxyzxyzDRTxyzDVTDVTCCAATCAGCATCAATTATCGCAC
FG/e/7	CATTACCGTGTACGCTGTAxyzxyzxyzDRTxyzxyzxyzCCAATCAGCATCAATTATCGCAC
