# Supplementary material for: High-Throughput Ligand Discovery Reveals a Sitewise Gradient of Diversity in Broadly Evolved Hydrophilic Fibronectin Domains
Source: PLoS One. 2015 Sep 18;10(9):e0138956. doi: 10.1371/journal.pone.0138956 (PMC4575168; doi:10.1371/journal.pone.0138956)
Supplement: S2 Table — Sequences are composed of standard nucleotides (ACGT), degenerate nucleotides (RYMKSWHBVDN), and a specialty codon mix (xyz) which uses the following nucleotide frequencies: 20% A, 15% C, 25% G, and 40% T at site 1, 50% A, 25% C, 15% G, and 10% T at site 2, and 0% A, 45% C, 10% G, and 45% T at site 3. Oligos are arranged by loop (BC, DE, FG), loop specific sublibraries, and amino acid length of the diversified region within the loop. (PDF) [file pone.0138956.s005.pdf]

S2 Table. Oligonucleotide DNA sequences used for constructing generation two library. Sequences are composed of standard nucleotides (ACGT), degenerate nucleotides (RYMKSWHBVDN), and a specialty codon mix (xyz) which uses the following nucleotide frequencies : 20% A, 15% C, 25% G, and 40% T at site 1, 50% A, 25% C, 15% G, and 10% T at site 2, and 0% A, 45% C, 10% G, and 45% T at site 3. Oligos are arranged by loop (BC, DE, FG), loop specific sublibraries, and amino acid length of the diversified region within the loop.

Loop/Sublib/Len (AA)	Oligo Sequence
BC/a/10	ACTCTCTGACTATTTCTTGGGACGCACCAxyzxyzxyzxyzDCTxyzGGATACCGTATCACCTACGGCGAAAC
BC/b/10	ACTCTCTGACTATTTCTTGGGACDMTYMTxyzxyzxyzxyzDCTxyzTATTACCGTATCACCTACGGCGAAAC
BC/c/10	ACTCTCTGACTATTTCTTGGGACGCACCAxyzxyzxyzxyzDCTxyzGGATACCGTATCACCTACGGCGAAAC
BC/d/10	ACTCTCTGACTATTTCTTGGGACDMTYMTxyzxyzxyzxyzDCTxyzTATTACCGTATCACCTACGGCGAAAC
BC/e/10	ACTCTCTGACTATTTCTTGGGACGCACCAxyzxyzxyzxyzDCTxyzGGATACCGTATCACCTACGGCGAAAC
BC/f/10	ACTCTCTGACTATTTCTTGGGACDMTYMTxyzxyzxyzxyzDCTxyzTATTACCGTATCACCTACGGCGAAAC
BC/g/10	ACTCTCTGACTATTTCTTGGGACGCACCAxyzxyzxyzxyzDCTxyzGGATACCGTATCACCTACGGCGAAAC
BC/h/10	ACTCTCTGACTATTTCTTGGGACDMTYMTxyzxyzxyzxyzDCTxyzTATTACCGTATCACCTACGGCGAAAC
BC/a/9	ACTCTCTGACTATTTCTTGGGACGCACCAxyzxyzxyzDCTxyzGGATACCGTATCACCTACGGCGAAAC
BC/b/9	ACTCTCTGACTATTTCTTGGGACDMTYMTxyzxyzxyzDCTxyzTATTACCGTATCACCTACGGCGAAAC
BC/c/9	ACTCTCTGACTATTTCTTGGGACGCACCAxyzxyzxyzDCTxyzGGATACCGTATCACCTACGGCGAAAC
BC/d/9	ACTCTCTGACTATTTCTTGGGACDMTYMTxyzxyzxyzDCTxyzTATTACCGTATCACCTACGGCGAAAC
BC/e/9	ACTCTCTGACTATTTCTTGGGACGCACCAxyzxyzxyzDCTxyzGGATACCGTATCACCTACGGCGAAAC
BC/f/9	ACTCTCTGACTATTTCTTGGGACDMTYMTxyzxyzxyzDCTxyzTATTACCGTATCACCTACGGCGAAAC
BC/g/9	ACTCTCTGACTATTTCTTGGGACGCACCAxyzxyzxyzDCTxyzGGATACCGTATCACCTACGGCGAAAC
BC/h/9	ACTCTCTGACTATTTCTTGGGACDMTYMTxyzxyzxyzDCTxyzTATTACCGTATCACCTACGGCGAAAC
BC/a/8	ACTCTCTGACTATTTCTTGGGACGCACCAxyzxyzDCTxyzGGATACCGTATCACCTACGGCGAAAC
BC/b/8	ACTCTCTGACTATTTCTTGGGACDMTYMTxyzxyzDCTxyzTATTACCGTATCACCTACGGCGAAAC
BC/c/8	ACTCTCTGACTATTTCTTGGGACGCACCAxyzxyzDCTxyzGGATACCGTATCACCTACGGCGAAAC

BC/d/8	ACTCTCTGACTATTTCTTGGGACDMTYMTxyzxyzDCTxyzTATTACCGTATCACCTACGGCGAAAC
BC/e/8	ACTCTCTGACTATTTCTTGGGACGCACCAxyzxyzDCTxyzGGATACCGTATCACCTACGGCGAAAC
BC/f/8	ACTCTCTGACTATTTCTTGGGACDMTYMTxyzxyzDCTxyzTATTACCGTATCACCTACGGCGAAAC
BC/g/8	ACTCTCTGACTATTTCTTGGGACGCACCAxyzxyzDCTxyzGGATACCGTATCACCTACGGCGAAAC
BC/h/8	ACTCTCTGACTATTTCTTGGGACDMTYMTxyzxyzDCTxyzTATTACCGTATCACCTACGGCGAAAC
DE/a/5	CGAGCCAGGAATTCACTGTTCCGGGAWMTWMTWMTWMTGCGACCATCAGCGGTCTGAAAC
DE/a/4	CGAGCCAGGAATTCACTGTTCCGGGAWMTWMTWMTGCGACCATCAGCGGTCTGAAAC
FG/a/11	CATTACCGTGTACGCTGTARSCDVTxyzRRCxyzxyzxyzxyzxyzTCAAACCCAATCAGCATCAATTATCGCAC
FG/a/10	CATTACCGTGTACGCTGTARSCDVTxyzRRCxyzxyzxyzxyzTCAAACCCAATCAGCATCAATTATCGCAC
FG/a/9	CATTACCGTGTACGCTGTARSCDVTxyzRRCxyzxyzxyzTCAAACCCAATCAGCATCAATTATCGCAC
FG/a/8	CATTACCGTGTACGCTGTARSCDVTxyzRRCxyzxyzTCAAACCCAATCAGCATCAATTATCGCAC
FG/a/7	CATTACCGTGTACGCTGTARSCDVTxyzRRCxyzTCAAACCCAATCAGCATCAATTATCGCAC
