# Supplementary material for: High-Throughput Ligand Discovery Reveals a Sitewise Gradient of Diversity in Broadly Evolved Hydrophilic Fibronectin Domains
Source: PLoS One. 2015 Sep 18;10(9):e0138956. doi: 10.1371/journal.pone.0138956 (PMC4575168; doi:10.1371/journal.pone.0138956)
Supplement: S3 Table — Library design can be guided by information regarding each position’s mutational tolerance and naturally evolved sequence to reduce the prevalence of overly destabilizing mutations as well as identifying structurally stabilizing mutations. Additionally, the chemical diversity found at the interfaces of well characterized natural binders, such as the complementarity determining regions (CDR) of antibodies, can be applied to protein scaffolds to accommodate for strong binding interactions. Here, a model for library design was built based on a linear combination of (A) computational stability, (B) natural homolog sequence frequency, and (C) CDR diversity input matrices. These three elements were weighted based on the (D) target exposure (i.e. proximity to the binding interface) and solvent exposed surface area (i.e. orientation and packing) of each site. (PDF) [file pone.0138956.s006.pdf]

S3 Table. Correlative Parametric Analysis of Amino Acid Distributions - Input Matrices. Library design can be guided by information regarding each position's mutational tolerance and naturally evolved sequence to reduce the prevalence of overly destabilizing mutations as well as identifying structurally stabilizing mutations. Additionally, the chemical diversity found at the interfaces of well characterized natural binders, such as the complementarity determining regions (CDR) of antibodies, can be applied to protein scaffolds to accommodate for strong binding interactions. Here, a model for library design was built based on a linear combination of (A) computational stability, (B) natural homolog sequence frequency, and (C) CDR diversity input matrices. These three elements were weighted based on the (D) target exposure (i.e. proximity to the binding interface) and solvent exposed surface area (i.e. orientation and packing) of each site.

A. Computational sitewise assessment of changes in stability (ΔΔG, kcal/mol) upon mutation. Conducted in FoldX.

	D23	A24	P25	A26	V27	T28	V29	R30	Y31	G52	S53	K54	S55	T56
A	0.34	-	0.28	-	(0.31)	(0.30)	1.67	0.23	0.06	1.70	(0.09)	0.73	(0.23)	0.26
C	0.37	0.29	0.42	0.01	(0.24)	(0.21)	1.28	0.86	(0.15)	3.38	(0.22)	0.86	0.07	0.03
D	-	0.73	0.72	(0.08)	(0.03)	(0.34)	3.28	0.65	1.51	5.39	0.00	0.93	(0.00)	0.97
E	0.18	0.64	0.56	(0.25)	(0.25)	(0.48)	3.24	0.39	0.29	5.24	(0.48)	0.55	(0.02)	(0.13)
F	(0.22)	0.51	0.75	(0.50)	(0.16)	(0.44)	4.17	0.18	(0.33)	6.16	(0.28)	(0.06)	(0.36)	(0.16)
G	1.11	0.35	0.15	0.21	0.02	(0.33)	2.54	0.76	0.34	-	0.20	1.70	0.05	0.75
H	0.59	0.73	1.50	0.10	0.33	(0.15)	4.09	0.91	0.75	6.14	0.14	0.96	0.03	0.54
I	0.30	0.98	0.64	(0.07)	(0.07)	(0.01)	(0.31)	0.81	0.40	11.73	(0.40)	0.95	0.16	(0.03)
K	(0.04)	0.13	0.49	(0.37)	(0.38)	(0.57)	3.01	(0.00)	(0.46)	4.76	(0.73)	-	(0.56)	(0.56)
L	(0.24)	0.38	0.02	(0.47)	(0.56)	(0.48)	0.82	(0.06)	(0.47)	6.31	(0.71)	(0.31)	(0.38)	(0.25)
M	(0.19)	0.04	(0.47)	(0.57)	(0.58)	(0.53)	0.81	0.07	(0.84)	4.41	(0.78)	(0.32)	(0.45)	(0.91)
N	0.35	0.32	0.52	(0.07)	(0.24)	(0.30)	2.13	0.64	0.65	4.25	(0.04)	0.75	(0.54)	0.63
P	2.35	(0.59)	-	(0.08)	1.28	(0.06)	3.89	2.73	2.37	3.52	0.28	3.58	(0.08)	1.71
Q	0.18	0.40	0.49	(0.26)	(0.25)	(0.35)	3.04	0.11	0.06	5.13	(0.39)	0.49	(0.29)	(0.10)
R	0.12	0.16	0.74	(0.32)	(0.22)	(0.51)	4.14	-	(0.40)	5.01	(0.53)	(0.12)	(0.83)	(0.51)
S	0.62	0.24	0.19	0.19	(0.27)	(0.23)	2.43	0.79	0.03	3.21	-	0.84	-	(0.17)
T	0.71	0.73	0.61	0.37	(0.18)	-	1.16	1.12	1.16	8.01	0.32	1.23	0.70	-
V	0.62	0.82	0.71	0.20	-	0.12	-	0.90	0.86	8.15	0.03	1.25	0.40	0.07
W	0.17	1.03	1.87	(0.50)	0.69	(0.19)	7.81	0.49	0.45	7.10	(0.17)	0.03	(0.08)	(0.14)
Y	(0.12)	0.45	1.36	(0.45)	0.01	(0.39)	6.36	0.29	-	6.31	(0.27)	0.00	(0.29)	(0.10)

	T76	G77	R78	G79	D80	S81	P82	A83	S84	S85	K86
A	(0.07)	0.46	0.45	0.37	0.02	(0.04)	(0.70)	(0.15)	(0.34)	(0.06)	0.34
C	(0.07)	0.38	0.68	0.44	0.04	0.06	(0.65)	0.04	(0.20)	(0.13)	0.32
D	1.17	0.68	0.60	0.15	-	(0.03)	(0.61)	(0.01)	0.05	(0.15)	0.51
E	0.58	0.72	0.17	0.27	(0.15)	(0.12)	(0.79)	(0.04)	0.02	(0.12)	0.23
F	(0.69)	(0.14)	0.03	0.11	(0.24)	(0.30)	(1.13)	(0.30)	(0.36)	(0.23)	(0.36)
G	0.45	-	0.70	-	(0.39)	(0.51)	(0.60)	0.14	0.17	0.41	0.19
H	0.97	0.91	0.44	0.40	0.13	0.27	(0.58)	0.04	0.31	0.49	0.31
I	(0.98)	0.68	0.47	1.00	0.62	0.30	(0.92)	(0.14)	(0.47)	0.03	0.79
K	(0.11)	0.15	(0.02)	(0.15)	(0.44)	(0.29)	(1.08)	(0.38)	(0.38)	(0.17)	(0.06)
L	(1.16)	0.06	(0.09)	0.01	(0.27)	(0.25)	(1.03)	(0.39)	(0.95)	(0.49)	(0.10)
M	(1.37)	(0.27)	0.05	0.14	(0.29)	(0.23)	(1.06)	(0.45)	(1.28)	(0.63)	(0.33)
N	0.38	0.24	0.45	0.12	(0.14)	(0.09)	(0.66)	(0.10)	(0.08)	(0.17)	0.41
P	1.30	1.43	0.64	2.26	1.17	1.70	-	0.53	2.03	0.40	1.32
Q	0.18	0.57	0.03	0.20	(0.13)	(0.20)	(0.93)	(0.29)	(0.31)	(0.16)	0.14
R	0.37	0.18	-	0.09	(0.23)	(0.22)	(0.76)	(0.23)	(0.55)	(0.09)	(0.00)
S	0.26	0.34	0.57	0.41	(0.18)	-	(0.36)	0.00	0.02	(0.08)	0.38
T	-	0.74	0.69	0.84	0.40	0.33	(0.38)	0.10	0.02	0.01	0.86
V	(0.55)	0.99	0.64	0.98	0.54	0.43	(0.57)	0.04	(0.19)	0.04	0.92
W	1.10	0.52	0.45	0.32	(0.08)	0.07	(0.89)	(0.16)	(0.19)	0.29	(0.20)
Y	0.03	0.03	0.10	0.19	(0.17)	(0.22)	(1.07)	(0.24)	(0.18)	0.06	(0.23)

B. Natural sequence frequency for the tenth type III domain of human fibronectin: pfam database summary. Below the full amino acid table, the four most frequent residues at each site are listed.

	V	S	D	V	P	R	D	L	E	V	V	A	A	T	P	T	S	L	L
	1	2	3	4	5	6	7	8	9	10	11	12	13	14	15	16	17	18	19
A	13.1%	12.6%	3.2%	28.6%	1.6%	4.5%	4.1%	2.1%	4.3%	15.0%	4.6%	11.2%	10.1%	2.0%	10.4%	1.7%	6.6%	11.2%	2.9%
C	0.5%	-	-	1.2%	-	-	-	-	-	2.2%	0.7%	0.6%	0.7%	-	0.6%	0.6%	1.0%	3.5%	0.5%
D	1.5%	0.8%	16.0%	-	0.7%	1.0%	13.4%	-	3.7%	0.9%	3.9%	15.8%	1.8%	4.3%	8.8%	13.9%	1.8%	-	3.1%
E	3.5%	1.1%	9.6%	0.8%	0.8%	6.9%	6.2%	-	13.3%	2.2%	5.6%	11.8%	3.1%	2.7%	8.6%	5.5%	5.1%	-	4.7%
F	0.8%	-	-	1.2%	-	1.6%	0.8%	5.0%	1.1%	5.1%	1.9%	0.7%	0.9%	0.9%	-	1.3%	1.2%	4.4%	2.5%
G	7.1%	22.2%	3.8%	3.0%	4.1%	8.8%	9.1%	-	1.6%	2.1%	2.7%	6.7%	4.7%	7.2%	5.0%	2.9%	4.5%	0.6%	1.2%
H	0.6%	-	1.6%	-	-	2.1%	1.9%	-	4.8%	-	1.5%	1.6%	1.1%	0.9%	2.3%	2.8%	1.9%	-	2.4%
I	2.7%	-	0.6%	7.4%	1.2%	2.1%	2.3%	11.8%	2.2%	11.7%	7.2%	1.4%	13.1%	0.8%	1.0%	1.0%	-	21.8%	4.3%
K	1.8%	3.6%	4.0%	1.9%	0.6%	8.5%	5.1%	-	11.5%	1.2%	4.7%	3.3%	3.9%	2.8%	11.7%	2.8%	3.3%	-	5.4%
L	13.2%	0.6%	1.3%	7.6%	0.8%	5.2%	1.4%	42.5%	1.5%	8.4%	6.5%	1.5%	7.8%	1.8%	1.1%	1.1%	-	19.6%	12.1%
M	3.9%	-	-	1.4%	-	1.2%	-	2.0%	0.9%	0.9%	1.4%	0.8%	1.3%	0.6%	0.9%	-	0.6%	5.6%	2.0%
N	0.8%	2.0%	4.3%	0.8%	1.2%	1.8%	25.6%	-	3.6%	1.0%	2.4%	11.1%	2.2%	8.5%	3.5%	11.8%	3.3%	-	3.9%
P	24.0%	14.3%	41.0%	11.1%	81.9%	1.8%	6.4%	8.7%	2.0%	2.0%	1.0%	4.5%	2.9%	0.6%	12.9%	-	-	-	0.6%
Q	2.1%	2.5%	5.1%	1.5%	0.9%	9.6%	3.4%	0.6%	9.2%	0.9%	2.3%	3.2%	2.4%	2.1%	3.3%	2.5%	3.5%	-	4.8%
R	1.9%	2.4%	2.7%	1.1%	-	14.0%	3.1%	-	11.1%	0.9%	3.6%	3.0%	3.7%	3.4%	4.9%	5.2%	2.7%	-	7.9%
S	4.3%	28.4%	3.1%	3.1%	2.0%	10.4%	9.0%	0.8%	9.2%	3.1%	14.0%	13.1%	6.8%	22.1%	16.2%	14.8%	45.8%	-	9.8%
T	4.4%	7.3%	1.5%	5.2%	1.5%	12.4%	5.4%	1.7%	14.0%	5.2%	18.7%	5.2%	7.4%	35.6%	4.9%	27.6%	13.4%	0.9%	21.5%
V	11.6%	0.7%	1.4%	22.8%	1.1%	5.7%	1.5%	21.7%	4.6%	34.8%	14.7%	3.3%	23.4%	1.5%	1.4%	2.6%	1.3%	28.7%	7.8%
W	-	-	-	-	-	-	-	-	-	0.7%	-	0.7%	1.3%	0.9%	0.8%	-	1.1%	1.0%	1.2%
Y	1.9%	-	-	0.6%	-	1.6%	-	-	0.7%	1.2%	2.2%	0.5%	1.1%	0.8%	1.2%	1.1%	1.9%	-	1.4%

Most Frequent

1	P (0.24)	S (0.28)	P (0.41)	A (0.29)	P (0.82)	R (0.14)	N (0.26)	L (0.43)	T (0.14)	V (0.35)	T (0.19)	D (0.16)	V (0.23)	T (0.36)	S (0.16)	T (0.28)	S (0.46)	V (0.29)	T (0.21)
2	L (0.13)	G (0.22)	D (0.16)	V (0.23)	G (0.04)	T (0.12)	D (0.13)	V (0.22)	E (0.13)	A (0.15)	V (0.15)	S (0.13)	I (0.13)	S (0.22)	P (0.13)	S (0.15)	T (0.13)	I (0.22)	L (0.12)
3	A (0.13)	P (0.14)	E (0.1)	P (0.11)	S (0.02)	S (0.1)	G (0.09)	I (0.12)	K (0.12)	I (0.12)	S (0.14)	E (0.12)	A (0.1)	N (0.08)	K (0.12)	D (0.14)	A (0.07)	L (0.2)	S (0.1)
4	V (0.12)	A (0.13)	Q (0.05)	L (0.08)	A (0.02)	Q (0.1)	S (0.09)	P (0.09)	R (0.11)	L (0.08)	I (0.07)	A (0.11)	L (0.08)	G (0.07)	A (0.1)	N (0.12)	E (0.05)	A (0.11)	R (0.08)

	I	S	W	D	A	P	A	V	T	V	R	Y	Y	R	I	T	Y	G	E
	20	21	22	23	24	25	26	27	28	29	30	31	32	33	34	35	36	37	38
A	1.9%	6.8%	-	2.6%	18.2%	2.2%	15.0%	9.2%	4.5%	6.9%	2.0%	3.9%	3.1%	2.1%	1.4%	2.9%	1.3%	4.8%	3.0%
C	-	1.0%	-	-	-	-	-	1.8%	-	0.7%	-	0.7%	1.0%	-	-	0.8%	1.8%	1.8%	0.7%
D	-	1.3%	-	16.5%	2.1%	3.7%	4.4%	6.4%	17.5%	6.2%	10.9%	2.2%	2.2%	4.2%	-	3.0%	0.6%	2.5%	7.2%
E	-	5.8%	0.5%	15.2%	6.2%	1.6%	9.4%	4.8%	5.6%	2.1%	6.3%	4.4%	2.9%	9.6%	0.6%	13.6%	1.6%	2.3%	23.9%
F	2.1%	0.6%	1.6%	-	-	-	-	3.0%	0.6%	4.0%	-	2.9%	8.9%	1.2%	1.0%	2.0%	6.0%	1.6%	0.9%
G	-	2.4%	0.5%	3.0%	4.2%	3.1%	13.5%	11.1%	4.8%	11.5%	18.1%	30.7%	4.5%	2.9%	1.3%	5.9%	1.1%	13.5%	-
H	-	2.7%	-	1.8%	1.5%	0.7%	1.2%	2.1%	1.6%	0.7%	1.9%	5.3%	1.4%	1.7%	-	1.5%	2.2%	2.4%	1.6%
I	16.1%	1.0%	-	0.8%	2.3%	-	2.2%	6.5%	1.9%	24.3%	1.7%	3.0%	3.7%	10.9%	37.3%	2.7%	3.4%	5.1%	4.0%
K	-	6.3%	-	8.9%	8.5%	1.6%	4.9%	3.0%	3.3%	0.6%	6.3%	3.4%	1.6%	9.5%	0.7%	4.6%	3.4%	8.8%	7.3%
L	44.5%	1.3%	0.7%	2.1%	2.6%	0.8%	4.1%	8.4%	1.8%	6.8%	5.1%	3.5%	2.3%	6.0%	17.6%	5.1%	5.4%	3.2%	4.4%
M	1.3%	1.3%	-	0.6%	1.1%	-	0.6%	1.6%	-	0.9%	0.5%	1.0%	-	1.0%	1.1%	0.9%	1.8%	1.3%	0.7%
N	-	4.7%	-	8.4%	2.2%	2.4%	5.3%	5.1%	12.1%	3.2%	5.4%	6.6%	1.7%	3.9%	0.5%	4.1%	0.9%	5.0%	2.2%
P	0.7%	1.1%	0.7%	3.7%	22.6%	73.0%	6.8%	5.6%	9.9%	3.0%	1.3%	1.2%	3.5%	3.7%	0.6%	0.8%	-	1.4%	8.5%
Q	-	6.5%	-	7.8%	2.5%	1.3%	4.3%	3.1%	3.7%	0.9%	6.8%	3.1%	1.2%	6.1%	0.6%	7.9%	2.1%	4.9%	5.3%
R	-	6.0%	-	3.8%	5.1%	0.6%	3.0%	2.3%	2.6%	0.6%	10.6%	3.1%	0.8%	15.5%	-	5.7%	4.5%	12.2%	7.1%
S	-	31.6%	-	9.6%	8.4%	4.4%	13.9%	8.0%	14.7%	2.5%	7.4%	11.5%	6.5%	3.7%	0.6%	10.4%	0.9%	9.1%	3.8%
T	0.6%	16.1%	-	12.7%	4.8%	1.4%	4.8%	4.0%	11.7%	2.7%	12.3%	1.9%	1.4%	5.8%	1.9%	17.6%	0.8%	5.5%	3.2%
V	29.7%	2.3%	-	1.4%	6.6%	1.9%	4.9%	8.4%	2.1%	20.4%	2.0%	2.2%	3.3%	9.2%	31.6%	5.6%	4.6%	6.7%	8.2%
W	0.9%	-	92.4%	-	-	-	-	-	-	-	-	0.9%	1.0%	0.6%	-	0.5%	4.8%	2.0%	1.0%
Y	-	0.8%	0.8%	-	-	-	0.8%	5.2%	0.8%	1.4%	0.5%	8.4%	48.4%	2.4%	1.8%	4.3%	52.4%	5.7%	6.4%

Most Frequent

1	L (0.45)	S (0.32)	W (0.92)	D (0.17)	P (0.23)	P (0.73)	A (0.15)	G (0.11)	D (0.17)	I (0.24)	G (0.18)	G (0.31)	Y (0.48)	R (0.15)	I (0.37)	T (0.18)	Y (0.52)	G (0.14)	E (0.24)
2	V (0.3)	T (0.16)	F (0.02)	E (0.15)	A (0.18)	S (0.04)	S (0.14)	A (0.09)	S (0.15)	V (0.2)	T (0.12)	S (0.12)	F (0.09)	I (0.11)	V (0.32)	E (0.14)	F (0.06)	R (0.12)	P (0.09)
3	I (0.16)	A (0.07)	Y (0.01)	T (0.13)	K (0.09)	D (0.04)	G (0.14)	V (0.08)	N (0.12)	G (0.11)	D (0.11)	Y (0.08)	S (0.06)	E (0.1)	L (0.18)	S (0.1)	L (0.05)	S (0.09)	V (0.08)
4	F (0.02)	Q (0.07)	P (0.01)	S (0.1)	S (0.08)	G (0.03)	E (0.09)	L (0.08)	T (0.12)	A (0.07)	R (0.11)	N (0.07)	G (0.04)	K (0.1)	T (0.02)	Q (0.08)	W (0.05)	K (0.09)	K (0.07)

	T 39	G 40	G 41	N 42	S 43	P 44	V 45	Q 46	E 47	F 48	T 49	V 50	P 51	G 52	S 53	K 54	S 55	T 56	A 57
A	6.3%	5.8%	6.1%	2.8%	6.5%	4.1%	6.5%	4.3%	7.4%	3.9%	4.9%	6.3%	5.4%	8.6%	6.5%	5.7%	4.3%	2.6%	16.7%
C	1.6%	1.5%	1.1%	0.6%	1.5%	-	1.0%	0.6%	1.3%	1.7%	1.5%	1.7%	0.7%	2.0%	1.2%	-	2.8%	1.2%	4.4%
D	3.8%	9.9%	7.5%	13.3%	6.2%	6.8%	2.2%	4.1%	8.0%	1.5%	4.9%	1.6%	7.6%	5.3%	7.8%	6.1%	4.8%	2.6%	1.4%
E	7.7%	5.0%	6.7%	12.4%	6.7%	11.4%	4.2%	8.7%	23.7%	2.8%	5.4%	2.8%	7.7%	3.1%	5.3%	12.6%	3.6%	9.0%	3.1%
F	1.6%	1.3%	1.1%	1.2%	1.0%	1.4%	3.3%	0.6%	0.5%	10.6%	0.5%	1.6%	0.6%	0.8%	0.8%	0.8%	1.6%	3.0%	6.4%
G	0.7%	19.6%	27.6%	6.1%	9.7%	5.4%	2.6%	4.9%	2.9%	2.4%	3.1%	1.5%	3.6%	32.9%	3.3%	2.2%	2.9%	1.6%	2.8%
H	0.9%	1.5%	1.2%	2.6%	1.5%	1.4%	0.6%	3.5%	2.1%	2.0%	1.4%	0.8%	1.7%	1.8%	2.6%	2.3%	1.8%	2.1%	4.2%
I	6.8%	3.3%	1.6%	1.8%	1.4%	2.2%	11.9%	1.6%	1.4%	8.4%	2.8%	12.1%	1.7%	2.8%	2.1%	2.2%	1.5%	2.3%	6.2%
K	8.4%	8.4%	5.6%	8.0%	7.4%	6.0%	2.7%	13.3%	7.2%	2.9%	5.2%	2.6%	5.9%	3.9%	4.2%	15.5%	2.6%	4.0%	3.4%
L	7.3%	4.0%	2.9%	2.8%	2.9%	2.8%	10.0%	4.2%	1.5%	11.7%	3.7%	11.2%	2.2%	2.2%	2.7%	3.9%	5.2%	3.6%	5.1%
M	2.1%	1.4%	1.2%	1.3%	0.8%	0.7%	3.1%	1.5%	0.9%	2.9%	1.2%	1.7%	0.7%	0.6%	0.6%	1.0%	1.3%	1.2%	2.0%
N	2.8%	6.6%	2.8%	14.5%	3.6%	2.7%	2.0%	4.7%	6.3%	3.4%	6.4%	1.9%	5.0%	5.7%	9.2%	4.8%	8.9%	5.1%	2.1%
P	2.9%	2.1%	2.7%	2.7%	1.3%	22.7%	0.8%	2.0%	2.2%	0.9%	3.6%	0.7%	28.2%	3.3%	3.8%	2.3%	3.0%	2.7%	2.1%
Q	2.6%	4.5%	3.5%	4.2%	5.2%	4.2%	1.2%	16.5%	3.9%	2.0%	4.6%	1.4%	3.2%	1.6%	3.0%	9.5%	3.3%	4.1%	2.5%
R	5.5%	5.3%	7.4%	5.9%	6.9%	4.1%	4.2%	9.3%	6.4%	3.6%	6.7%	2.4%	3.3%	3.3%	4.0%	10.4%	5.6%	5.5%	2.1%
S	9.2%	8.2%	9.1%	6.2%	18.8%	6.9%	4.3%	7.1%	6.7%	3.3%	12.6%	3.3%	8.4%	8.1%	18.9%	4.8%	17.1%	17.2%	4.1%
T	14.9%	3.3%	5.2%	6.5%	10.8%	7.1%	8.9%	5.2%	8.1%	4.0%	23.6%	6.9%	7.5%	5.8%	16.9%	7.7%	24.0%	23.3%	5.5%
V	11.8%	4.1%	2.7%	2.4%	3.7%	2.1%	21.5%	2.2%	5.2%	14.5%	5.9%	36.2%	5.3%	5.5%	4.7%	5.0%	3.3%	4.5%	11.5%
W	0.6%	1.1%	1.4%	1.3%	1.7%	6.2%	5.5%	3.1%	2.9%	8.9%	1.0%	1.1%	-	1.9%	1.0%	1.9%	0.6%	2.1%	1.1%
Y	2.5%	3.3%	2.5%	3.4%	2.5%	1.5%	3.5%	2.4%	1.3%	8.7%	1.0%	2.2%	0.8%	0.8%	1.3%	0.9%	1.8%	2.3%	13.2%
Most Frequent																			
1	T (0.15)	G (0.2)	G (0.28)	N (0.15)	S (0.19)	P (0.23)	V (0.21)	Q (0.16)	E (0.24)	V (0.14)	T (0.24)	V (0.36)	P (0.28)	G (0.33)	S (0.19)	K (0.15)	T (0.24)	T (0.23)	A (0.17)
2	V (0.12)	D (0.1)	S (0.09)	D (0.13)	T (0.11)	E (0.11)	I (0.12)	K (0.13)	T (0.08)	L (0.12)	S (0.13)	I (0.12)	S (0.08)	A (0.09)	T (0.17)	E (0.13)	S (0.17)	S (0.17)	Y (0.13)
3	S (0.09)	K (0.08)	D (0.07)	E (0.12)	G (0.1)	T (0.07)	L (0.1)	R (0.09)	D (0.08)	F (0.11)	R (0.07)	L (0.11)	E (0.08)	S (0.08)	N (0.09)	R (0.1)	N (0.09)	E (0.09)	V (0.12)
4	K (0.08)	S (0.08)	R (0.07)	K (0.08)	K (0.07)	S (0.07)	T (0.09)	E (0.09)	A (0.07)	W (0.09)	N (0.06)	T (0.07)	D (0.08)	T (0.06)	D (0.08)	Q (0.1)	R (0.06)	R (0.06)	F (0.06)

	T	I	S	G	L	K	P	G	V	D	Y	T	I	T	V	Y	A	V	T
	58	59	60	61	62	63	64	65	66	67	68	69	70	71	72	73	74	75	76
A	1.9%	3.0%	3.3%	3.3%	1.0%	3.5%	6.7%	6.1%	5.3%	3.8%	1.7%	2.7%	3.0%	2.1%	2.1%	3.2%	45.5%	2.2%	2.7%
C	0.8%	0.9%	0.5%	-	-	-	0.7%	-	1.6%	-	-	0.7%	0.9%	1.1%	-	1.1%	1.1%	1.5%	1.1%
D	4.0%	2.9%	6.3%	11.7%	0.9%	3.6%	2.4%	7.2%	-	11.5%	-	3.3%	1.0%	2.1%	1.3%	0.9%	1.8%	0.5%	2.2%
E	5.9%	1.0%	7.3%	4.1%	1.0%	10.9%	9.9%	3.2%	3.1%	21.7%	1.9%	11.0%	1.3%	6.4%	3.3%	3.1%	6.0%	3.8%	5.9%
F	1.1%	6.1%	1.1%	0.8%	3.2%	1.1%	1.4%	3.0%	1.1%	0.5%	4.4%	1.3%	24.9%	0.8%	0.8%	11.4%	-	8.2%	3.2%
G	0.6%	-	3.1%	38.1%	-	0.9%	1.5%	41.5%	0.8%	1.6%	1.4%	2.6%	1.8%	2.5%	2.8%	1.2%	5.5%	0.9%	1.1%
H	2.0%	-	2.5%	2.6%	-	2.3%	0.9%	2.1%	2.2%	2.0%	2.9%	1.8%	-	1.8%	0.5%	2.9%	1.0%	1.2%	1.1%
I	4.3%	22.4%	0.9%	0.7%	4.5%	5.1%	1.9%	0.5%	4.2%	1.1%	1.2%	2.5%	18.2%	0.9%	12.5%	3.5%	1.1%	13.3%	2.0%
K	6.7%	0.9%	7.4%	4.3%	0.5%	16.6%	3.9%	2.4%	4.5%	7.4%	1.8%	8.4%	0.9%	6.1%	1.6%	5.5%	3.9%	1.1%	4.3%
L	5.7%	21.9%	1.7%	1.2%	71.2%	5.8%	1.9%	3.0%	11.6%	4.8%	4.2%	6.0%	13.9%	2.8%	15.3%	8.2%	2.2%	12.6%	2.7%
M	1.3%	2.0%	0.8%	0.5%	1.8%	1.6%	0.6%	0.6%	2.1%	0.8%	0.8%	0.9%	2.8%	0.7%	1.6%	1.8%	0.5%	1.9%	0.9%
N	4.6%	1.1%	5.3%	13.6%	0.7%	4.0%	3.0%	10.9%	2.7%	6.6%	1.2%	6.9%	0.9%	4.9%	2.4%	1.7%	2.6%	1.1%	19.9%
P	2.5%	0.8%	4.2%	1.9%	0.5%	1.4%	45.8%	-	-	2.7%	1.0%	1.9%	0.6%	1.2%	1.4%	1.1%	3.9%	1.0%	1.7%
Q	3.5%	1.5%	4.7%	2.7%	0.7%	8.3%	2.6%	1.6%	3.2%	6.2%	0.9%	6.4%	-	5.4%	0.9%	5.2%	4.1%	0.9%	4.1%
R	6.5%	0.8%	5.2%	3.0%	1.3%	9.5%	2.1%	2.3%	5.3%	4.5%	2.2%	5.7%	-	21.1%	1.1%	8.7%	4.8%	0.9%	5.4%
S	6.1%	0.8%	17.6%	5.4%	0.8%	7.9%	5.0%	7.5%	5.7%	9.2%	1.1%	10.5%	0.7%	11.7%	1.0%	4.7%	7.5%	1.6%	10.1%
T	31.6%	2.3%	23.2%	2.4%	3.3%	10.4%	6.0%	2.6%	31.5%	11.6%	1.4%	20.9%	1.5%	22.4%	1.9%	4.9%	5.0%	3.0%	24.3%
V	9.4%	28.2%	2.4%	1.3%	6.3%	5.3%	2.3%	0.8%	13.1%	2.2%	2.0%	3.9%	21.3%	1.5%	46.6%	4.4%	1.9%	34.7%	2.9%
W	-	-	0.7%	-	-	-	-	0.7%	-	0.6%	0.5%	-	-	1.1%	-	2.0%	-	0.8%	0.5%
Y	1.1%	2.2%	1.9%	1.7%	0.9%	1.4%	1.1%	3.1%	0.7%	-	68.5%	2.0%	4.5%	3.3%	2.0%	24.5%	0.8%	8.7%	3.9%
Most Frequent																			
1	T (0.32)	V (0.28)	T (0.23)	G (0.38)	L (0.71)	K (0.17)	P (0.46)	G (0.41)	T (0.31)	E (0.22)	Y (0.69)	T (0.21)	F (0.25)	T (0.22)	V (0.47)	Y (0.25)	A (0.46)	V (0.35)	T (0.24)
2	V (0.09)	I (0.22)	S (0.18)	N (0.14)	V (0.06)	E (0.11)	E (0.1)	N (0.11)	V (0.13)	T (0.12)	F (0.04)	E (0.11)	V (0.21)	R (0.21)	L (0.15)	F (0.11)	S (0.07)	I (0.13)	N (0.2)
3	K (0.07)	L (0.22)	K (0.07)	D (0.12)	I (0.04)	T (0.1)	A (0.07)	S (0.08)	L (0.12)	D (0.12)	L (0.04)	S (0.1)	I (0.18)	S (0.12)	I (0.13)	R (0.09)	E (0.06)	L (0.13)	S (0.1)
4	R (0.06)	F (0.06)	E (0.07)	S (0.05)	T (0.03)	R (0.09)	T (0.06)	D (0.07)	S (0.06)	S (0.09)	H (0.03)	K (0.08)	L (0.14)	E (0.06)	E (0.03)	L (0.08)	G (0.05)	Y (0.09)	E (0.06)

	G	R	G	D	S	P	A	S	S	K	P	I	S	I	N	Y	R	T
	77	78	79	80	81	82	83	84	85	86	87	88	89	90	91	92	93	94
A	18.6%	6.7%	8.8%	1.1%	8.1%	5.6%	42.2%	10.0%	6.6%	2.6%	8.7%	11.4%	7.0%	6.1%	4.8%	4.2%	8.4%	9.4%
C	0.7%	-	1.1%	0.7%	2.0%	0.8%	1.1%	2.6%	1.0%	-	-	1.6%	0.7%	1.0%	-	-	-	-
D	2.6%	0.6%	0.6%	22.8%	0.7%	1.3%	-	1.7%	4.3%	1.3%	2.3%	0.7%	1.2%	3.4%	8.8%	2.2%	2.1%	1.0%
E	5.3%	7.6%	1.5%	17.5%	0.8%	5.3%	2.7%	10.2%	2.4%	19.6%	4.3%	1.2%	1.3%	5.3%	7.8%	10.5%	14.6%	1.8%
F	4.5%	2.0%	4.6%	3.5%	3.7%	2.2%	2.6%	2.5%	1.7%	0.5%	-	7.1%	-	4.0%	-	14.1%	2.5%	0.6%
G	14.6%	-	49.6%	1.1%	9.1%	2.5%	8.6%	13.9%	23.6%	7.0%	8.3%	4.0%	32.0%	6.1%	36.5%	4.7%	2.3%	2.8%
H	1.7%	3.0%	0.8%	1.1%	0.8%	1.2%	-	1.9%	0.6%	2.2%	0.8%	0.5%	-	-	0.6%	3.4%	1.0%	-
I	2.9%	2.3%	2.1%	4.3%	3.4%	6.5%	5.8%	1.8%	1.3%	0.7%	2.4%	13.2%	-	14.8%	-	1.3%	-	1.1%
K	4.9%	13.9%	0.8%	2.8%	3.9%	5.6%	1.0%	3.4%	1.0%	21.3%	1.7%	2.0%	0.8%	1.3%	2.7%	4.1%	9.0%	1.0%
L	2.8%	3.0%	2.3%	4.0%	2.5%	4.0%	6.7%	2.7%	1.2%	1.9%	0.6%	14.2%	-	15.4%	0.7%	4.4%	1.6%	2.1%
M	1.5%	1.1%	1.2%	0.6%	1.0%	3.2%	2.3%	0.8%	-	0.6%	-	3.7%	-	2.5%	-	1.2%	-	0.5%
N	7.6%	1.7%	5.5%	13.4%	6.4%	1.7%	1.4%	5.0%	15.6%	10.2%	3.3%	1.1%	2.5%	0.9%	11.5%	1.1%	1.0%	0.7%
P	3.4%	-	1.0%	-	-	23.6%	-	-	-	0.5%	55.1%	0.6%	-	2.0%	3.5%	11.1%	19.0%	6.4%
Q	3.3%	5.2%	1.4%	4.7%	3.0%	5.4%	1.6%	4.9%	2.0%	9.7%	1.8%	1.2%	1.2%	1.4%	2.6%	2.2%	5.4%	0.8%
R	1.9%	31.2%	0.8%	5.7%	7.4%	8.2%	2.6%	5.7%	1.4%	12.1%	1.3%	0.8%	1.0%	1.3%	1.4%	1.9%	16.3%	0.5%
S	7.6%	1.7%	3.5%	1.7%	19.4%	2.0%	1.2%	15.8%	21.7%	2.8%	2.9%	0.7%	45.9%	2.5%	14.9%	5.0%	7.6%	55.5%
T	3.0%	2.5%	3.7%	3.0%	11.4%	4.3%	1.6%	7.7%	7.9%	3.0%	3.0%	2.6%	4.5%	4.2%	2.8%	1.6%	3.0%	13.0%
V	6.8%	5.4%	3.1%	7.2%	12.0%	12.7%	13.6%	4.9%	4.7%	1.3%	1.8%	24.1%	-	22.6%	-	3.5%	0.8%	1.4%
W	0.6%	-	0.5%	-	-	-	0.7%	-	-	-	-	1.4%	-	2.1%	-	9.5%	2.3%	-
Y	5.7%	10.9%	7.1%	3.7%	4.3%	3.3%	3.2%	4.0%	2.1%	2.1%	0.6%	7.8%	-	2.6%	-	13.6%	1.8%	-
Most Frequent																		
1	A (0.19)	R (0.31)	G (0.5)	D (0.23)	S (0.19)	P (0.24)	A (0.42)	S (0.16)	G (0.24)	K (0.21)	P (0.55)	V (0.24)	S (0.46)	V (0.23)	G (0.37)	F (0.14)	P (0.19)	S (0.55)
2	G (0.15)	K (0.14)	A (0.09)	E (0.18)	V (0.12)	V (0.13)	V (0.14)	G (0.14)	S (0.22)	E (0.2)	A (0.09)	L (0.14)	G (0.32)	L (0.15)	S (0.15)	Y (0.14)	R (0.16)	T (0.13)
3	N (0.08)	Y (0.11)	Y (0.07)	N (0.13)	T (0.11)	R (0.08)	G (0.09)	E (0.1)	N (0.16)	R (0.12)	G (0.08)	I (0.13)	A (0.07)	I (0.15)	N (0.11)	P (0.11)	E (0.15)	A (0.09)
4	S (0.08)	E (0.08)	N (0.06)	V (0.07)	G (0.09)	I (0.07)	L (0.07)	A (0.1)	T (0.08)	N (0.1)	E (0.04)	A (0.11)	T (0.04)	A (0.06)	D (0.09)	E (0.11)	K (0.09)	P (0.06)

C. Complementarity distribution mimicking CDRH3 of antibodies (CDR'), modified from previous work (Hackel et al., 2010).

CDR' amino acid frequency distribution:

A	0.06
C	0.05
D	0.11
E	0.02
F	0.03
G	0.04
H	0.06
I	0.02
K	0.02
L	0.02
M	0.00
N	0.09
P	0.04
Q	0.01
R	0.03
S	0.13
T	0.05
V	0.03
W	0.01
Y	0.17
Z	0.03

Nucleotide composition of codon design:

A1	C1	G1	T1	A2	C2	G2	T2	A3	C3	G3	T3
0.20	0.15	0.25	0.40	0.50	0.25	0.15	0.10	0.00	0.45	0.10	0.45

D. Target exposure and solvent exposed surface area (SASA) for the BC, DE, and FG loops of fibronectin.

**Position**	**Target Exposure**	**SASA**
D23	0.68	0.35
A24	0.90	0.32
P25	0.70	0.10
A26	0.79	0.75
V27	0.69	0.57
T28	0.84	0.75
V29	0.46	0.03
R30	0.90	0.49
Y31	0.37	0.43
G52	0.54	0.12
S53	0.94	0.83
K54	0.68	0.64
S55	0.85	0.41
T56	0.93	0.48
T76	0.39	0.08
G77	0.60	0.48
R78	0.79	0.81
G79	0.72	0.77
D80	0.70	0.74
S81	0.54	0.69
P82	0.59	0.76
A83	0.76	0.81
S84	0.52	0.54
S85	0.61	0.14
K86	0.58	0.88
