# Supplementary material for: High-Throughput Ligand Discovery Reveals a Sitewise Gradient of Diversity in Broadly Evolved Hydrophilic Fibronectin Domains
Source: PLoS One. 2015 Sep 18;10(9):e0138956. doi: 10.1371/journal.pone.0138956 (PMC4575168; doi:10.1371/journal.pone.0138956)
Supplement: S4 Table — Conserved framework positions with regions or sites having low diversity, as is the case with conserved framework positions, require additional considerations during sample preparation to ensure a high level of accuracy during the MiSeq run. The inclusion of variable length degenerate sequence (N4-8) at 5’ and 3’ ends allow the conserved sites to be offset. Based on TruSeq guidelines, adapter indices are designed to have balanced G/T and C/A content, the following 6 adapter index tags were selected: AD005,6,12,14,18,19. Schematic below demonstrates the two-step PCR used for amplicon library construction. Colored regions of schematic indicate (from left to right) TruSeq universal adapter (red), target primer (cyan), gene of interest (gray), reverse target primer (green), multiplex primer 2.0 (yellow), Illumina index (blue), and Illumina PCR primer (purple). Table at bottom lists individual sequences with item names corresponding to PCR schematic. (PDF) [file pone.0138956.s007.pdf]

S4 Table. Illumina primer design. Conserved framework positions with regions or sites having low diversity, as is the case with conserved framework positions, require additional considerations during sample preparation to ensure a high level of accuracy during the MiSeq run. The inclusion of variable length degenerate sequence ($N_{4\text{-}8}$) at 5' and 3' ends allow the conserved sites to be offset. Based on TruSeq guidelines, adapter indices are designed to have balanced G/T and C/A content, the following 6 adapter index tags were selected: AD005,6,12,14,18,19. Schematic below demonstrates the two-step PCR used for amplicon library construction. Colored regions of schematic indicate (from left to right) TruSeq universal adapter (red), target primer (cyan), gene of interest (gray), reverse target primer (green), multiplex primer 2.0 (yellow), Illumina index (blue), and Illumina PCR primer (purple). Table at bottom lists individual sequences with item names corresponding to PCR schematic.

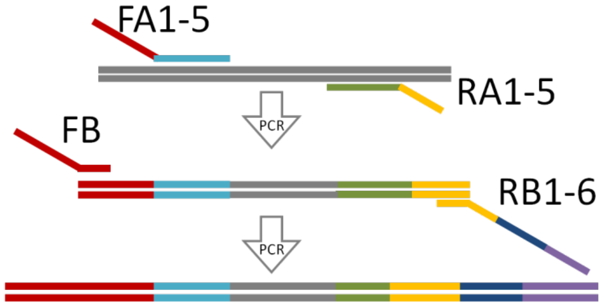

Item Name	Length	Sequence
RB1	59	caagcagaagacggcatacgagat**CACTGT**gtgactggagttcagacgtgtgctcttcc
RB2	59	caagcagaagacggcatacgagat**ATTGGC**gtgactggagttcagacgtgtgctcttcc
RB3	59	caagcagaagacggcatacgagat**TACAAG**gtgactggagttcagacgtgtgctcttcc
RB4	59	caagcagaagacggcatacgagat**TTTCAC**gtgactggagttcagacgtgtgctcttcc
RB5	59	caagcagaagacggcatacgagat**GGAACT**gtgactggagttcagacgtgtgctcttcc
RB6	59	caagcagaagacggcatacgagat**GCGGAC**gtgactggagttcagacgtgtgctcttcc
FB	51	aatgatacggcgaccaccgagatctacactctttccctacacgacgctctt
RA1	49	GTTCAGACGTGTGCTCTTCCGATCTNNNNATAAGCTTTTGTTCGGATCC
RA2	50	GTTCAGACGTGTGCTCTTCCGATCTNNNNNATAAGCTTTTGTTCGGATCC
RA3	51	GTTCAGACGTGTGCTCTTCCGATCTNNNNNNATAAGCTTTTGTTCGGATCC
RA4	52	GTTCAGACGTGTGCTCTTCCGATCTNNNNNNNATAAGCTTTTGTTCGGATCC
RA5	53	GTTCAGACGTGTGCTCTTCCGATCTNNNNNNNNATAAGCTTTTGTTCGGATCC
FA1	51	TTTCCCTACACGACGCTCTTCCGATCTNNNNACTACGCTCTGCAGGCTAGT
FA2	52	TTTCCCTACACGACGCTCTTCCGATCTNNNNNACTACGCTCTGCAGGCTAGT
FA3	53	TTTCCCTACACGACGCTCTTCCGATCTNNNNNNACTACGCTCTGCAGGCTAGT
FA4	54	TTTCCCTACACGACGCTCTTCCGATCTNNNNNNNACTACGCTCTGCAGGCTAGT
FA5	55	TTTCCCTACACGACGCTCTTCCGATCTNNNNNNNNACTACGCTCTGCAGGCTAGT

TruSeq guidelines PDF available online: http://support.illumina.com/content/dam/illumina-support/documents/myillumina/f5f619d3-2c4c-489b-80a3-e0414baa4e89/truseq_dna_sampleprep_guide_15026486_c.pdf
